# Understanding protein import in diverse non-green plastids

**DOI:** 10.3389/fgene.2023.969931

**Published:** 2023-03-16

**Authors:** Ryan Christian, June Labbancz, Bjorn Usadel, Amit Dhingra

**Affiliations:** ^1^ Department of Horticulture, Washington State University, Pullman, WA, United States; ^2^ Department of Horticultural Sciences, Texas A&M University, College Station, TX, United States; ^3^ Institute for Biology I, Aachen, Germany

**Keywords:** plastid, non-green plastid, transit peptide, protein import, protein translocation, proteomics

## Abstract

The spectacular diversity of plastids in non-green organs such as flowers, fruits, roots, tubers, and senescing leaves represents a Universe of metabolic processes in higher plants that remain to be completely characterized. The endosymbiosis of the plastid and the subsequent export of the ancestral cyanobacterial genome to the nuclear genome, and adaptation of the plants to all types of environments has resulted in the emergence of diverse and a highly orchestrated metabolism across the plant kingdom that is entirely reliant on a complex protein import and translocation system. The TOC and TIC translocons, critical for importing nuclear-encoded proteins into the plastid stroma, remain poorly resolved, especially in the case of TIC. From the stroma, three core pathways (cpTat, cpSec, and cpSRP) may localize imported proteins to the thylakoid. Non-canonical routes only utilizing TOC also exist for the insertion of many inner and outer membrane proteins, or in the case of some modified proteins, a vesicular import route. Understanding this complex protein import system is further compounded by the highly heterogeneous nature of transit peptides, and the varying transit peptide specificity of plastids depending on species and the developmental and trophic stage of the plant organs. Computational tools provide an increasingly sophisticated means of predicting protein import into highly diverse non-green plastids across higher plants, which need to be validated using proteomics and metabolic approaches. The myriad plastid functions enable higher plants to interact and respond to all kinds of environments. Unraveling the diversity of non-green plastid functions across the higher plants has the potential to provide knowledge that will help in developing climate resilient crops.

## 1 Introduction

The plastids have descended from an ancient cyanobacterial symbiont acquired by a mitochondria-harboring eukaryotic host between 1.5 and 1.2 billion years ago (McFadden and Van Dooren, 2004; Yoon et al., 2004; Falcón et al., 2010). The plastid of modern land plants retains a small genome from its cyanobacterial ancestor, ranging from 120 to 160 kb in size, containing about 90 protein-coding genes mainly related to photosynthesis, transcription, and translation (Sugiura, 1992; Green, 2011). This stands in contrast with modern cyanobacteria which have 3,000-7,500 potential genes (Kaneko et al., 1996; Meeks et al., 2001); despite this enormous reduction in the plastid genome, the plastids of higher plants still contain 2000–5000 unique proteins, with between 4 and 11% of the nuclear genome being plastid-targeted (Ajjawi et al., 2010; Christian et al., 2020b). Horizontal gene transfer from the plastid genome to the nuclear genome has contributed to this situation, with the nuclear genome of *Arabidopsis thaliana* containing roughly 4500 genes which are of cyanobacterial origin, and some are not targeted to the plastids (Martin et al., 2002). This shift is explained by the phenomenon of Muller’s ratchet, in which non-recombining, asexually-reproducing genomes gradually accumulate irreversible mutations, thus favoring horizontal transfer to the sexually-reproducing nuclear genome (Muller, 1964; Lynch and Blanchard, 1998; Martin and Herrmann, 1998), a process further favored by the free-radical rich, mutation-inducing environment within the plastids (Allen and Raven, 1996) and selective pressure for rapid reproduction (Selosse et al., 2001). The reduction of the size and gene content by transfer the plastid and the nuclear genome interdependent. Experiments assessing DNA transfer from plastids to nuclei estimate the rate of transfer to be as high as 1 in 16,000 in pollen grains (Huang et al., 2003) to as low as 1 in 5 million in vegetative cells (Stegemann et al., 2003), suggesting this transfer may have occurred rapidly after endosymbiosis.

For biochemical functionality of the plastid to be maintained through the export of genes to the nuclear genome, a means of selectively importing gene products into the plastid became necessary (Zimorski et al., 2014). This selectivity comes from “transit peptides,” a term to distinguish chloroplast and mitochondrial targeting sequences from the more uniform signal peptides used for trafficking to the endoplasmic reticulum and secretion pathways (Chua and Schmidt, 1979). Transit peptides have become incredibly diverse, with negligible sequence homology and significant variance in length, spanning between 13 and 146 residues (Mackenzie, 2005). It is hypothesized that this secretory machinery was converted into import machinery by reversing the direction of peptide transport (McFadden, 1999), resulting in the TOC and TIC translocons, a complex of several proteins responsible for the vast majority of the import into the plastid. This import apparatus is another feature which defines the plastid and sets it apart from less developed endosymbiotic relationships (Martin and Herrmann, 1998). The research has revealed a highly dynamic and multifaceted import system that is vastly more complex than cognate translocons of other organelles.

## 2 Evolution of the plastid

The first plastid-bearing organisms were the ancestors of modern Glaucophyta, Rhodophyta, and Viridiplantae (Gould et al., 2008). The plastids of glaucophytes and algae are largely photosynthetic, much like chloroplasts in higher plants in function, with a shared genome of just over 100 genes (Figueroa-Martinez et al., 2019). The plastids of glaucophytes retain a peptiodgylcan wall much like their cyanobacterial ancestor (Keeling, 2010), while the plastids of Rhodophyta possess phycobilins (Gabrielson et al., 1990), enabling the capture of red, orange, and green wavelengths of light at a greater efficiency than chlorophyll (Reviewed in Simkin et al., 2022). Secondary and tertiary endosymbiosis events, the result of organisms taking on the plastids of other plastid containing organisms and forming new symbioses, have led to plastids being spread to other eukaryotes including Apicomplexa, Heterokonta, Dinoflagellata, and Eugenida (Gould et al., 2008). These too, are largely photosynthetic, possessing chlorophylls and carotenoids to harvest light energy.

While most of these plastid-bearing lineages possess photosynthetic plastids comparable to the chloroplasts of land plants in function, some have developed non-green and non-photosynthetic plastids. Apicomplexa and Helicosporidium are notable in being parasites with non-photosynthetic plastids—in Apicomplexa they are termed apicoplasts (Maréchal and Cesbron-Delauw, 2001; de Koning and Keeling, 2006). The plastids of both have biosynthetic roles in common with their photosynthetic counterparts despite their loss of photosynthesis, including fatty acid, amino acid, and terpenoid biosynthesis (Maréchal and Cesbron-Delauw, 2001; Lim and McFadden, 2010; Sheiner et al., 2013). Rhodelphis, a sister group to Rhodophyta, has non-green plastids which lack a plastid genome entirely, and are primarily responsible for heme biosynthesis (Gawryluk et al., 2019). Even among the green algae, some lineages have developed non-photosynthetic plastids which most closely resemble the amyloplasts of land plants but has a reduced proteome of only about 300 proteins (Fuentes-Ramírez et al., 2021). Unlike the non-green plastids of land plants, most of these non-green plastids represent reduced forms of the original photosynthetic plastids acquired over a billion years ago.

A second known primary endosymbiotic event in the genus *Paulinella* has occurred much more recently (140–90 million years ago), in which an amoeboid formed an endosymbiosis with what was most likely a cyanobacteria to form a photosynthetic plastid called a chromatophore, strongly resembling a photosynthetic chloroplast (Delaye et al., 2016; Singer et al., 2017). While the chromatophore has undergone a reduction of the plastid genome, possessing a genome about 1/3 the size of the smallest sequenced cyanobacteria (Nowack et al., 2008), and horizontal gene transfer from the chromatophore genome to the nuclear genome is underway (Zhang et al., 2017), this process has not yet progressed to the same degree as in plastids derived from the first primary endosymbiotic event. Less than 1% of the genes in *Paulinella chromatophora* were obtained from horizontal gene transfer from the chromatophore (Nowack et al., 2011), as opposed to more than 6% in plants and algae (Price et al., 2012), and the chromatophore genome remains about 5–10 times larger than comparable photosynthetic plastids (Selosse et al., 2001; Nowack et al., 2008). Another recently discovered organism, *Pseudoblepharisma tenue*, a ciliate which has formed an endosymbiosis with both photosynthetic purple bacteria and green algae, is another example of a budding primary endosymbiosis. The purple bacteria endosymbionts have a reduced genome about half the size of their closest known relatives and have lost genes essential for nitrogen and sulfur metabolism, as well as the use of hydrogen sulfide as an electron donor for photosynthesis, while retaining genes necessary for independent aerobic respiration (Muñoz-Gómez et al., 2021). This endosymbiosis is less developed than that of the chromatophoresin *Paulinella* and plastids in plants, however, with no evidence of translocons for protein import and a fairly independent metabolism, including aerobic respiration*.* These organisms possibly represent different stages of the endosymbiosis process which the plastids of land plants underwent over a billion years ago; they provide both a historic insight into the past of more familiar organisms, while also demonstrating that primary endosymbiotic events might not be as rare as previously thought. It is possible that modern plastids are the result of multiple endosymbiotic events and multiple phases of horizontal gene transfer resulting in both a plastid and nuclear genome of multiple origins, with a corresponding proteome resulting from multiple endosymbiotic events (Howe et al., 2008). Bacteria other than cyanobacteria have contributed extensively to the genome of plastids as well, with more than 6% of the plastid proteome coming from non-cyanobacterial prokaryotes (Qiu et al., 2013). The multi-sourced origins of the plastid genome and proteome fits the “shopping bag model” of plastids, where the physical compartment of the plastid arose from a single event, but the contents cannot be attributed to a single origin (Howe et al., 2008). Whether this is responsible for some of the flexibility of plastid function and the plastid’s role in non-photosynthetic processes deserves further study.

Land plants, the focus of this review, angiosperms in particular, owing to their highly differentiated tissues and complex life cycles involving flowering and fruiting, have developed a wide variety of plastid morphotypes, which can differ within an organism between tissues and developmental stages. Diverse non-green plastid morphotypes developed early in the evolutionary history of land plants to adapt to challenges of life on land. All vascular plant lineages possess gravitropic amyloplasts to guide root development, and amyloplasts also appear in the reproductive cells of primitive vascular plants as a means of storage (Bell, 1986; Cao et al., 2012; Zhang et al., 2019). Chromoplasts appear in all seed plant lineages as well, suggesting a common origin at least 300 million years ago (Whatley, 1985; Jiao et al., 2011), likely a result of the importance of photoprotection and interactions with animals for all seeded plants. Angiosperms have the greatest amount of studied non-green plastid morphotypes, reflecting both their dominance within most ecosystems from the Cretaceous onwards (Coiffard et al., 2012), as well as their varied roles in flowering and fruiting.

Plastid morphotype variants in Angiosperms are well-described by microscopy, including the archetypical chloroplast and pre-chloroplastic proplastids, chloro-chromo-amyloplast, pigmented chromoplasts, biochemically-active leucoplasts, and starch-storing amyloplasts (Wise, 2006; Solymosi and Keresztes, 2013; Schaeffer et al., 2017). Within a species, plastids can rapidly change form in response to developmental or environmental cues, as exemplified in the etioplast to chloroplast transition in seedlings and the chloroplast to chromoplast transition, which is well documented in tomato (Muraki et al., 2010). New forms of plastids are still being discovered, including tannosomes in grape, which export phenolic precursors to the vacuole (Brillouet et al., 2013), dessicoplasts or xeroplasts, which protect plastids during extreme drought stress (Tuba et al., 1994; Ingle et al., 2008), and phenyloplasts, which accumulate a single large osmiophilic vesicle that stores phenol glucosides in vanilla orchid (Brillouet et al., 2014). In developing apple peel, novel hybrid plastids displaying both chromoplast and leucoplast characteristics arise in the epidermal cell layer, while hybrid chloroplast/amyloplasts predominate in collenchymal tissue (Solymosi and Keresztes, 2013; Schaeffer et al., 2017). These morphological and biochemical changes are mediated by the regulation of plastid gene expression and differential import of nuclear-encoded plastid-targeted genes. Plastid morphogenesis and differentiation is a complex and multifaceted process that alters the quantity and abundance of nuclear-encoded proteins as well as the transcription and translation rate of genes in the chloroplast genome (Liebers et al., 2017). Understanding the basis of extreme functional and metabolic heterogeneity in diverse plastid morphotypes will help in identifying mechanisms that will aid in developing climate resilient crops.

## 3 Components of protein import into the plastids

### 3.1 Transit peptides

The first step in importing nuclear genome-encoded proteins into the plastid is the presence of a suitable transit peptide at its N-terminus. Transit peptide import specificity enables differentiation of the plastid from the surrounding cytosol and contributes to the development of varying plastid morphotypes. Since the first ancestral transit peptides, a considerable degree of evolution and diversification has occurred, primarily driven by random insertions, deletions, and alternative splicing in duplicated genes (Christian et al., 2020a). The first transit peptides are hypothesized to have originated from ancient cyanobacterial virulence factors. Evidence to support this hypothesis includes proteins in the plastid translocon that have homologs which are hypothesized to be cyanobacterial virulence factors (Reumann et al., 1999; Reumann and Keegstra, 1999) and the GTPase-modulating properties and membrane-destabilizing properties of plastid transit peptides (Nicolay et al., 1994; Van ’t Hof and de Kruijff, 1995; Pinnaduwage and Bruce, 1996). This membrane destabilizing property also supports another hypothesis that transit peptides originate from antimicrobial peptides, with which transit peptides share a considerable degree of sequence similarity (Garrido et al., 2020).

The sequence similarity is limited, however, and developing a universal model for identifying transit peptides has proven difficult due to their sequence and length variability. The “homology block” hypothesis was the first proposed model that described most transit peptides containing three separate degenerate domains, sometimes encoded by individual exons (Karlin-Neumann and Tobin, 1986). More recent analysis has shown that chloroplast transit peptides are more accurately subdivided into seven subgroups. However, only half of the known transit peptides can be confidently organized into these groups (Dong et al., 2008). A unifying hypothesis, the “multi-selection, multi-order” or “M&M” model, attempts to reconcile these observations by using a more relaxed model of transit peptide construction in which domain organization is spatially unconstrained and allows for duplicate or optional sequence motifs (Li and Teng, 2013). This model is quite close to that of promoter elements, with a few core motifs and a multitude of cis-acting factors altering import efficiency; the creation of synthetic transit peptides using only a few critical motifs successfully localized to chloroplasts, lending credit to this “M&M” model of plastid protein import (Lee et al., 2015)⁠.

**FIGURE 1:**
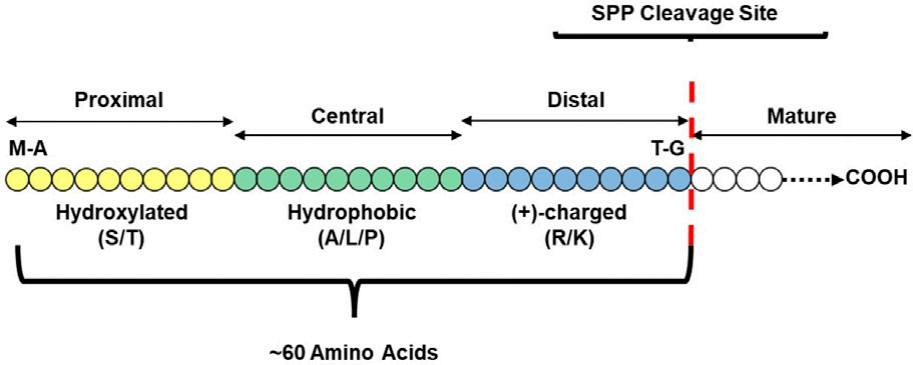
Basic Transit Peptide Structural Model. The three major domains required for a functional transit peptide include a hydroxylated N-terminus (N-domain) for binding to cytosolic and stromal chaperones, a hydrophobic, uncharged central domain responsible for bridging the outer and inner envelopes, and a positively-charged C-terminus that interacts with TOC GTPases to stabilize early translocation intermediates and ultimately trigger full translocation. Some elements may be repeated or be present in only some transit peptides, such as acidic residues at the C-terminus that may confer selectivity for certain TOC GTPases.

While transit peptide structure is incredibly heterogeneous, some generalizations can still be made, depicted in Figure 1. Certain residues are more abundant, with hydrophobic and hydroxylated residues generally enriched (von HEIJNE et al., 1989; Patron and Waller, 2007; Christian et al., 2020a); in Arabidopsis, serine is the most abundant residue in transit peptides at 19.3%, followed by leucine (10.4%), proline (7.3%), alanine (7.1%), and threonine (6.9%) (Zhang and Glaser, 2002; Zybailov et al., 2008; Li and Chiu, 2010). The first 20 amino acids at the N terminal are generally uncharged, often beginning with a methionine-alanine residue pair and ending in either glycine or proline (Claros et al., 1997). The presence of arginine in this region results in localization to the mitochondria, while replacing these arginine residues with serine or alanine results in plastid localization; small changes in amino acid identity in this region can radically alter import efficiency (Lee et al., 2019)⁠. Hsp70 binding sites, which occur in 95% of *Arabidopsis* transit peptides, are enriched in this region; 70% occur in this N-terminal region (Chotewutmontri et al., 2012; Chotewutmontri and Bruce, 2015). The central region is a spacer region dominated by small amino acids (Claros et al., 1997; Holbrook et al., 2016) and must be long enough to bridge the inner and outer membranes of the plastid, enabling interaction with both TIC and TOC (Chotewutmontri et al., 2012; Chen and Li, 2017). While the central third is a spacer region, some motifs in this region can regulate import efficiency (Chu et al., 2020). The C terminal region is enriched in charged amino acids (Claros et al., 1997; Christian et al., 2020a), with two motifs predominating: the FGLK (in Rubisco small subunit and ferredoxin) and dipositive motifs (Pilon et al., 1995). The overall biochemical structure of the preprotein also influences import efficiency and may necessitate changes in transit peptide structure. For example, peptide sequences for intermembrane proteins significantly reduce import efficiency, requiring longer spacer regions and more prolines in the transit peptide to compensate (Inaba and Schnell, 2008; Rolland et al., 2016). The complex nature of transit peptide structure and import into the plastid makes their identification *via* predictive algorithms an ongoing challenge.

Differences in transit peptide sequence and import efficiency are essential components in differentiating plastid morphotypes. The presence of a twin-positive motif (two positively charged amino acids in succession) in a transit peptide is predictive of import into root leucoplasts over leaf chloroplasts (Chu et al., 2020), and the same motif increases specificity for older chloroplasts over younger chloroplasts (Teng et al., 2012); this motif works in tandem with the RVSI motif in some peptides (Chu et al., 2020). Transit peptide targeting has been proposed to be a factor in the development of the unique single-cell C4 phenotype in Bienertia, with mutations of specific amino acids resulting in a reduction of import to peripheral plastids to a greater degree than central plastids (Wimmer et al., 2017). Transit peptides appear to be species-specific in many cases, with Arabidopsis unable to localize preproteins containing rice transit peptides to the plastid, and expression of preproteins containing Arabidopsis transit peptides in rice led to a loss of plastid-type specificity, likely arising from differences in the TOC/TIC translocons in each species (Eseverri et al., 2020). Differences in the average amino acid frequency of transit peptides exist between monocots and eudicots. For example, higher alanine and glycine frequency in monocots and higher serine and asparigine frequency in eudicots, respectively (Christian et al., 2020a), further complicates creating a universal model of transit peptide structure. This limited number of motifs studied and the varying specificity between species hinders prediction of protein import which could be used to understand plastid proteomes in non-model species and hinders predictions which could make precision plastid engineering possible.

### 3.2 TOC components

The TOC (translocon on the outer chloroplast membrane) complex is the first translocon encountered as preproteins are imported into the plastid, working together with transit peptides as the primary regulators in import of gene products from the nuclear genome. The core TOC complex of TOC159, TOC34, and TOC75 were first isolated and described in the early 1990s (Kessler et al., 1994; Schnell et al., 1994). In addition to these core components, myriad transient-interacting subunits including TOC64, Hsp70, Hsp90, and 14-3-3 participate in TOC functionality.

TOC75 is a *ß*-barrel protein, homologous with the OEP80 gene family in bacteria, which is thought to form the channel of the translocase with a pore size of 30-35Å and is capable of importing folded proteins (Inoue and Potter, 2004; Ganesan and Theg, 2019). New evidence in green algae suggests TOC75 forms a hybrid *ß*-barrel pore in conjunction with TOC120 to conduct preproteins (Jin et al., 2022), though the prevalence of this configuration is unknown. TOC33 and TOC34, the two major TOC34 paralogs, represent the smaller GTPase receptors. TOC33 consists of a GTPase “G-domain” and a short C-terminal segment facing the intermembrane space (Seedorf et al., 1995). The C-terminal segment is essential for biogenesis of TOC33 (Li and Chen, 1997), while the G-domain exhibits GTP hydrolysis activity and can form homodimers in its GDP-bound state (Sun et al., 2002; Yeh et al., 2007; Wiesemann et al., 2019). TOC33 forms homodimers and heterodimers with TOC159 (Sun et al., 2002; Jelic et al., 2003; Yeh et al., 2007; Wiesemann et al., 2019). Conserved arginine residues in the G-domain are likely to be central to the receptor activity of TOC33 and TOC34, and putatively act as a GTPase-activating protein (GAP) for bound preproteins (Sun et al., 2002; Reddick et al., 2007; Koenig et al., 2008). TOC159, the larger GTPase receptor, has multiple isoforms encoded by separate genes, including TOC90, TOC120, TOC132, and TOC159 in Arabidopsis. All isoforms, with the exception of TOC90 which lacks an A-domain, have three major domains: a GTPase (G-) domain, a C-terminal membrane (M-) domain, and a hypervariable N-terminal acidic (A-) domain (Hiltbrunner et al., 2001; Jackson-Constan and Keegstra, 2001), which is hypothesized to be responsible for specificity of different TOC isoforms (Inoue et al., 2010). TOC159 functions as a GTPase-activated switch (Richardson et al., 2018), and the force required for translocation comes as a pulling mechanism from the combined action of the Ycf2 complex or cpHsp70/Hsp90C/Hsp93; the identity of the motor protein remains controversial as both complexes associate with the TOC/TIC translocons and show ATPase activity (Shi and Theg, 2010; Su and Li, 2010; Inoue et al., 2013; Liu et al., 2014; Huang et al., 2016; Kikuchi et al., 2018; Li et al., 2020). Like TOC33, TOC159 can also form dimers using similarly conserved arginine residues (Yeh et al., 2007). Mutants that have defects in both binding and hydrolysis have impaired rates of translocation (Agne et al., 2009), but mutants which bind but not hydrolyze GTP increase translocation rates (Wang et al., 2008), suggesting that GTP-bound TOC159 is the translocation-active form.

**FIGURE 2:**
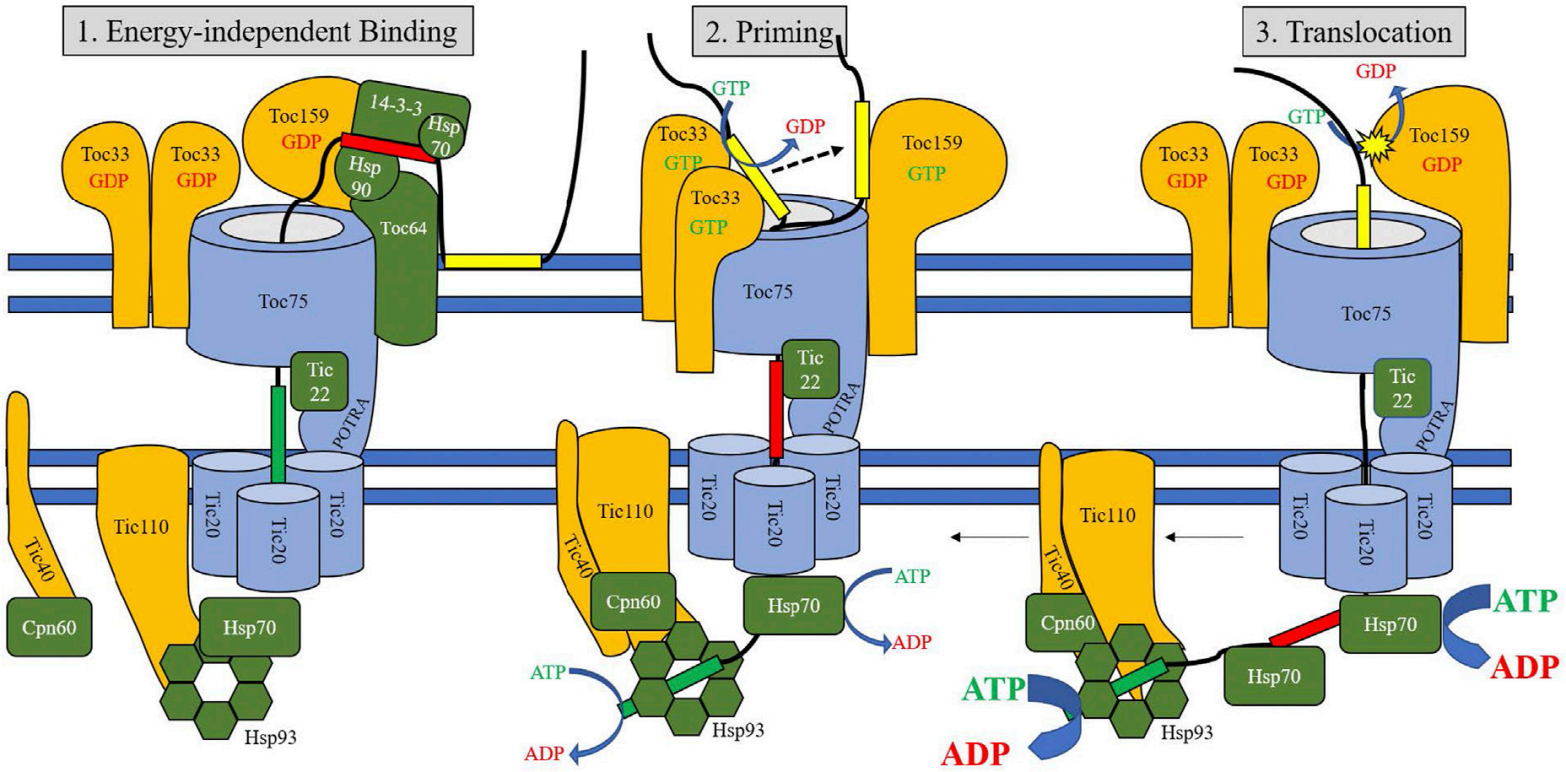
Model of TOC/TIC translocation. The three stages of translocation are represented in three panels (A–C), corresponding to Energy-Independent Binding, Priming, and Translocation stages. Initial stages (1) involve binding of the central transit peptide domain to cytosolic chaperones, and binding of the proximal domain to TIC22 and the TO75 POTRA domains. Transition to the early translocation intermediate stage (2) involves translocation of the proximal domain across the inner membrane and binding to stromal chaperones, while the distal domain is stabilized by TOC GTPases. Triggering the final stage of translocation (3) involves a final GTPase cycle for primed transit peptides, followed by strong ATPase activity of stromal Hsp70 and other chaperones to pull the preprotein into the stroma. The N-terminal motifs of the transit peptide are represented in green, the central motifs in red, and the C-terminal motifs in yellow. Proteins shown in blue represent pore or channel proteins, proteins shown in orange are receptor or scaffold proteins, and proteins shown in green are chaperones.

Transiently interacting soluble proteins also support preprotein trafficking to membrane-bound TOC components. Hsp90 acts as a molecular chaperone, binding preproteins in the cytosol and delivering them to TOC64 (Qbadou et al., 2006); TOC64 is capable of recognizing a guidance complex composed of a preprotein, Hsp70, and 14-3-3 proteins in order to facilitate translocation (Sohrt and Soll, 2000). TOC64 efficiently binds Hsp70.1 and Hsp90 using clamp-type tetratricopeptide repeats (Qbadou et al., 2006; Schweiger et al., 2013), serving as an intermediate receptor in this pathway before passing preproteins to TOC33 (Qbadou et al., 2006). The absence of functional TOC64 has been observed to create varying effects, from little significant change (Aronsson et al., 2007), to impaired translocation efficiency, photosynthetic activity, and salt tolerance (Sommer et al., 2013). Additionally, *toc33/toc64* double mutants have lower levels of TOC75 protein despite increased toc75 expression, suggesting it has a role in turnover and stabilization of the TOC complex (Sommer et al., 2013). The alternative chaperone-mediated route for chloroplast import involves binding of a 14-3-3/Hsp70 complex to phosphorylated serine and threonine residues in cTPs, which is regulated by the cytosolic kinases STY8, STY17, and STY46 (May and Soll, 2000; Lamberti et al., 2011a). Replacement of serine residues with non-phosphorylatable alanine did not show a decrease in import efficiency, however, indicating this chaperone activity is likely independent of phosphorylation (Holbrook et al., 2016). Binding to the 14-3-3 complex increases efficiency of import up to 5-fold by the formation of a “guidance complex” (May and Soll, 2000), but as in the Hsp90 pathway, disruption of binding interaction does not cause mistargeting (Nakrieko et al., 2004). STY kinase expression is linked to the transition from etioplast to chloroplast, suggesting that this chaperone-assisted route is similarly important during periods of high protein import demand (Lamberti et al., 2011b). It should be emphasized that preproteins can also travel unaided to the TOC complex, albeit with reduced import efficiency. Figure 2 displays the TOC components assembled into a translocon.

### 3.3 Regulation of TOC

Regulation of protein import at TOC is primarily based on alternative GTPase isoforms which regulate selectivity of preproteins; a summary of regulation points for TOC subunits is provided in Table 1. Non-redundant mutant phenotypes for the TOC33/34 (Jarvis et al., 1998; Gutensohn et al., 2000; 2004) and TOC90/120/132/159 (Bauer et al., 2000; Kubis et al., 2004) receptor gene families indicate that there are specialized TOC isoforms for certain classes of preproteins (Jarvis et al., 1998; Bauer et al., 2000; Ivanova et al., 2004; Kessler and Schnell, 2009). Selectivity is largely mediated by the hypervariable A-domain of the TOC159 GTPase family, removal of this domain greatly impairs selectivity (Inoue et al., 2010; Dutta et al., 2014). The A-domain does not bind transit peptides directly, but exchange of the A-domains between Toc159 homologs is sufficient to transfer the respective preprotein selectively (Inoue et al., 2010; Dutta et al., 2014), suggesting that the A-domain relies on an exclusion mechanism, perhaps based on steric hindrance or electrostatic repulsion (Smith et al., 2004; Inoue et al., 2010). The distinct TOC complex isoforms are hypothesized to reduce competition between protein classes such that the housekeeping and the photosynthetic proteins can be simultaneously imported through separate complexes (Kessler and Schnell, 2006). TOC159 is associated with photosynthetic proteins and has higher expression in leaves, while TOC132 and TOC120 are functionally redundant, constitutively expressed paralogs which import housekeeping proteins (Bauer et al., 2000; Ivanova et al., 2004; Kubis et al., 2004; Smith et al., 2004).

**TABLE 1:** Regulation Points of TOC/TIC. All current literature on regulation of protein import by formation of disulfide bridges, protein-protein interactions, proteolysis, phosphorylation, and ligand binding are summarized. Where known, the effect of each component on import is indicated.

Category	Component	Cofactors/Ligands	Effect on import	References
Disulfide Bridge	Toc64		Decrease	Sohrt and Soll (2000)
Disulfide Bridge	Tic110	Tic40	Decrease	Stahl et al. (1999), Balsera et al. (2009)
Disulfide Bridge	Toc75	Toc33, Toc159	Decrease	Seedorf and Soll (1995), Stengel et al. (2009)
Disulfide Bridge	Tic55		Unknown	Bartsch et al. (2008), Balsera et al. (2009)
Disulfide Bridge	Tic110-Tic40		Unknown	Stahl et al. (1999)
Protein-Protein Interaction	Tic32	Calmodulin	Decrease	Chigri et al. (2006)
Protein-Protein Interaction	Tic62	FNR	Decrease	Stengel et al. (2008)
Proteolysis	Toc159	Ubiquitin/SP1 E3 Ligase	Decrease	See Ling and Jarvis, 2015 and Ling and Jarvis, 2016
Phosphorylation	Toc159	KOC1	Increase	Zufferey et al. (2017)
Phosphorylation		SnRK2		
Phosphorylation	Toc159	OEK70 (KOC1?)	Decrease (no data)	Fulgosi and Soll (2002)
Phosphorylation	Toc33		Decrease	Sveshnikova et al. (2000), Jelic et al. (2002)
Phosphorylation	Toc33	OEK98	Decrease	Fulgosi and Soll (2002)
Ligand Binding	Tic55	Thioredoxin	Unknown	Bartsch et al. (2008)
Ligand Binding	Tic110	Thioredoxin	Decrease	Balsera et al. (2009)
Ligand Binding	Tic62	NADPH	Increase	Stengel et al. (2008), Balsera et al. (2009)
Ligand Binding	Tic32	NADPH	Increase	Chigri et al. (2006), Balsera et al. (2009)

Permanent changes in protein translocation specificity are mediated by Suppressor of PPI1 Locus 1 (SP1), an E3 ligase in the outer envelope which regulates turnover of TOC34, TOC75, and TOC159 homologs (Ling et al., 2012). SP1 is activated by stress and plays a pivotal role in stress tolerance by depleting TOC, limiting the import of photosynthesis related proteins, therefore decreasing photooxidative stress (Ling and Jarvis, 2015). Turnover of TOC receptors may also enable a rapid transition to chromoplasts, leucoplasts, or other plastid morphotypes (Reiland et al., 2011; Barsan et al., 2012), with experimental evidence showing higher SP1 expression accelerates the ripening process in tomato fruit and *sp1* mutants in Arabidopsis undergo highly inefficient developmental transitions (Reiland et al., 2011; Barsan et al., 2012; Ling et al., 2012; Ling et al., 2021)⁠. The specificity of TOC isoforms for different classes of preproteins likely makes them key regulators of the plastid proteome and morphotype, meaning major physiological changes must be accompanied by a similarly major refresh in TOC proteins. SP2 and CDC48 proteins are needed to extract the SP1 ubiquinated proteins from the outer membrane, and SP2 deficient plants have similar physiological disorders and delays as SP1 deficient plants (Ling et al., 2019). SP2 has sequence homology with TOC75 and likely forms a channel to assist in the extraction of membrane proteins (Shanmugabalaji and Kessler, 2019). The action of the three proteins SP1, SP2, and CDC48 are collectively the chloroplast-associated protein degradation (CHLORAD) pathway (Ling et al., 2019; Shanmugabalaji and Kessler, 2019), which also appears to function within the plastid, retrotranslocating plastid proteins to the cytosol for degradation by the 26S proteasome (Sun et al., 2022).

In addition to altering specificity, post-translational modification alters the rate of protein translocation. At least 12 sites on the A-domain of TOC159 can be phosphorylated (Demarsy et al., 2014); kinases phosphorylating this domain include casein kinase II (CKII) (Agne and Kessler, 2010), kinase of the outer chloroplast 1 (KOC1) (Zufferey et al., 2017), and sucrose non-fermenting 1-related protein kinase 2 (SnRK2) (Wang et al., 2013), all of which stimulate import. SnRK2 phosphorylates in the presence of ABA, which interrupts PP2C’s inhibition of SnRK2, perhaps explaining the impairment in chloroplast translocation and accumulation of larger, more abundant, and more highly-pigmented chromoplasts in ABA-deficient mutants (Galpaz et al., 2008; Zhong et al., 2010; Soon et al., 2012), as TOC159 selectively imports photosynthetic proteins. TOC33/34 are also proposed to be phosphorylated *in vivo* by both a soluble kinase and by a 98 kDa membrane-bound kinase OEK98 (Sveshnikova et al., 2000; Fulgosi and Soll, 2002; Jelic et al., 2002; 2003). Some reports find that GTP binding and preprotein binding activities of TOC33 are significantly inhibited by phosphorylation (Sveshnikova et al., 2000; Jelic et al., 2003), but phosphomimic and phosphoknockout residue mutants do not have a significant phenotype (Aronsson et al., 2006). The disparity between these observations remains unresolved, although it is possible that the partial redundancy of TOC34 can sufficiently complement the mutant phenotypes. Redox state is also a potent regulator of preprotein translocation efficiency, part of which is mediated by the TOC complex (Hirohashi and Nakai, 2000; Küchler et al., 2002; Stengel et al., 2008). Disulfide bridge formation has been observed in all the major TOC protein components in oxidizing conditions, which induces supramolecular crosslinking and decreases import efficiency (Seedorf et al., 1995; Stengel et al., 2009; Sjuts et al., 2017). This mechanism may serve to lock TOC in a translocation-incompetent state, preventing import into senescent or stressed chloroplasts until conditions improve (Stengel et al., 2009).

### 3.4 TIC channel components

The TIC (translocon on the inner chloroplast membrane) complex serves as a second regulatory point in the process of preprotein import, though it is less well understood than the TOC complex. The identity of the core TIC channel has been a subject of debate due to observed association with the TIC translocon for both TIC110 and TIC20. For many years, TIC110 was seen as a candidate channel protein because it is one of the most abundant proteins of the inner membrane (Kessler and Schnell, 2006), it demonstrated preprotein-dependent channel activity (Heins et al., 2002; Balsera et al., 2009), and it is found to some degree in TOC/TIC supercomplexes (Kouranov et al., 1998; Chen and Li, 2017). Evidence against this view has mounted, however. Less than 5% of TIC110 is associated with TOC complexes based on chromatography experiments, which would be unlikely for a channel protein if TOC and TIC are contiguous as crosslinking experiments suggest (Kouranov et al., 1998). TIC110 crystal structure indicates that it is unlikely to form a channel *in vivo* (Tsai et al., 2013), and it does not form tight associations with other TIC candidate components, and previous experiments may have overestimated the permanence of interactions between TIC110 and other TIC proteins (Kikuchi et al., 2013; Nakai, 2015a)⁠. TIC110 is also absent in the apicoplasts of Apicomplexans, which although simpler than higher plant plastids, retain a functional TOC/TIC translocon (Nakai, 2015a). The prior TIC110-centric model has been supplanted by a model for a core 1-MDa TIC complex comprised of a TIC20 channel supported by Tic21, TIC56, TIC100, and TIC214/YCF1 subunits (Kikuchi et al., 2009; Kikuchi et al., 2013), with TIC40 and TIC110 functioning instead as chaperone-recruiting scaffolds (Kikuchi et al., 2009; Inoue et al., 2013), or as scaffolds jutting into the stroma for proteins exiting the TIC complex, to be released by TIC40 (Inaba et al., 2003; Chou et al., 2006)⁠. This model is supported by TIC20’s is similarity in sequence and topology to TIM17/23, the inner membrane channel proteins of mitochondria (Inaba and Schnell, 2008; Kasmati et al., 2011).

Several observations render the TIC20 model incomplete. TIC20 is between 8 and 100-fold less abundant than TIC110 (Kovács-Bogdán et al., 2011), although it is still present at a ratio of 1:2.5 between TIC20 and TOC75, which could be expected if a single TIC channel serves four to seven TOC channels (Kikuchi et al., 2013). The most significant problem for the Tic20 hypothesis lies in inconsistent genetic evidence for its supporting subunits. YCF1 is absent from the plastid genome of grasses, glaucophytes, rhodophytes, and parasitic plants, while TIC56 and TIC100 are also absent outside of higher plants (Nakai, 2015b; de Vries et al., 2015). Furthermore, high levels of import are observed for a subset of proteins when TIC56 or YCF1 are inhibited (Köhler et al., 2015; Bölter and Soll, 2017). Mutant tic100 Arabidopsis plastids import less than one-third of the protein of wild type plants, and the abundance of 1-MDa translocation complex are reduced by more than one-half, however (Loudya et al., 2022). TIC21 is suggested to be an essential translocon component (Teng et al., 2006; Kikuchi et al., 2009), while it has also been characterized an iron transporter and is phylogenetically related to cyanobacterial permeases (Duy et al., 2007); later studies indicated that it does not co-purify with the TOC/TIC translocation complex (Kikuchi et al., 2013; Nakai, 2015a). Due to inconsistencies in both the TIC110 and TIC20 channel models, many authors have instead suggested that there are two independent TIC channels. One hypothesis posits that TIC110 serves as the general translocon pore while TIC20 imports a specialized subset (Kovács-Bogdán et al., 2011), and another argues for a redox-active TIC110 channel and a redox-independent TIC20 channel (Stengel et al., 2009). Finally, others suggest that TIC110 and TIC20 operate as independent but equally important channels (Demarsy et al., 2014; Bölter and Soll, 2016). The current evidence supports a TIC20-centered channel, but questions regarding the compositional inconsistency of the TIC channel remains to be addressed.

An additional TIC protein, TIC236, projects a 230 kDa domain into the intramembrane space and leads to the development of TOC/TIC supercomplex; knockout mutants are embryonically lethal (Chen et al., 2018) and the import of TIC22 (which uses the TOC-TIC translocon for localization to the intermembrane space) was greatly hampered in knockdown mutants of TIC236 (Chuang et al., 2021). This physical link between the TOC and TIC complexes appears to be necessary for translocation, and its association with TIC20 may lend support to it being the core TIC channel. Figure 2 displays these TIC components assembled as a translocon.

### 3.5 Regulation of TIC

While confusion regarding the identity of the TIC translocon hampers understanding of its regulation, it appears regulation at TIC is tied to the physiological status of the individual plastid rather than isoform composition as in TOC; a summary of regulation points for TIC subunits is provided in Table 1. Formation of disulfide bridges is common in TIC subunits, including intramolecular bridges in TIC110, TIC40, TIC55, and supramolecular bridges between TIC40 and TIC110 (Balsera et al., 2010); some of these disulfide bridges are regulated by stromal thioredoxins (Bartsch et al., 2008). As in the case of TOC, disulfide bridge formation arrests active translocation, while reducing agents and dithiols are effective at relieving this inhibition (Stengel et al., 2009). Additionally, protein-protein interactions regulate the import rate through TIC. The redox regulon of TIC32, TIC55, and TIC62 negatively regulate TIC110 and TIC40 based on redox status and other physiological conditions (Caliebe et al., 1997; Küchler et al., 2002; Hörmann et al., 2004). TIC32 is an NADPH-dependent dehydrogenase that binds competitively to NADPH and calmodulin, thus integrating both redox and calcium levels to fine-tune protein translocation affinity or efficiency (Küchler et al., 2002; Chigri et al., 2005). TIC62 is also an NADPH-dependent dehydrogenase, but it binds with ferredoxin:NADP(H) oxidoreductase (FNR) instead of calmodulin (Küchler et al., 2002; Stengel et al., 2008). While NADPH-bound, TIC62 decreases translocation, but upon FNR binding, it dissociates into a soluble complex in the stroma (Chigri et al., 2006; Stengel et al., 2008). TIC55 is a Rieske-type monooxygenase which was initially described to have effects on translocation (Caliebe et al., 1997), but a lack of definitive phenotype in the mutants has cast doubt on that role (Boij et al., 2009; Chou et al., 2018). However, observed roles in chlorophyll breakdown and dark-induced senescence may instead indicate a specific regulatory function in senescent plastids (Hauenstein et al., 2016; Chou et al., 2018). For an overview of regulatory points in TOC/TIC translocation, refer to Table 1.

### 3.6 Preprotein processing

Once successful translocation is initiated, the transit peptide must be cleaved to produce a mature, stable protein or reveal secondary transit signals to route proteins to the inner envelope, thylakoid membrane, or the lumen (Zhong et al., 2003). Initial cleavage of the transit peptide is performed by stromal processing peptidase (SPP) (Richter and Lamppa, 1998; 1999), but the new N-terminus of the protein is further polished in most cases in a process called “maturation”, primarily describing two post-translational modifications: removal of the N-terminal methionine by methionine aminopeptidase (Apel et al., 2010) and N-terminal acetylation by AT2G39000/AtNAA70 (Dinh et al., 2015). The N-terminal residue is a major determinant of protein stability in the plastid, following the “N-end” rule (Bachmair et al., 1986; Apel et al., 2010; Rowland et al., 2015; van Wijk, 2015). Artificial peptides starting with glutamic acid, methionine, and valine are especially stable in chloroplasts, while peptides starting with asparagine, cysteine, glutamine, histidine, isoleucine, proline, and threonine are unstable (Apel et al., 2010). Once removed by SPP, free transit peptides are membrane-seeking and can penetrate membranes, disrupting membrane potential and decoupling redox status (Nicolay et al., 1994; Pinnaduwage and Bruce, 1996; Wieprecht et al., 2000) making their quick degradation after initial cleavage into free amino acids essential for plastid functionality. Free transit peptides are degraded in a stepwise manner according to their size: 20-65 residue peptides by presequence proteases 1 and 2, 11-20 residue peptides by organellar oligopeptidase, and 3-5 residue peptides by metalloprotease M17-20 (Teixeira et al., 2017).

Plastid-targeted locations other than the stroma require further trafficking. Proteins bound for the inner envelope typically contain canonical N-terminal transit peptides that function identically to stromal transit peptides (Cline and Henry, 1996; Lee et al., 2017), while preproteins bound for the thylakoid membrane or lumen have a secondary transit peptide called a “thylakoid transfer domain” downstream of the SPP cleavage site (Smeekens et al., 1986; de Boer and Weisbeek, 1991).

## 4 Non-canonical import

While the vast majority of plastid-targeted proteins appear to use the TOC/TIC translocons (Row and Gray, 2001), a small subset of proteins, up to about 11% (Armbruster et al., 2009), are targeted and inserted *via* three major alternative routes: the outer envelope pathway, the inner membrane pathway, and the vesicular pathway.

The outer envelope proteins are a major group of non-canonically-imported proteins, with the sole known exception of TOC75-III following classical TOC import, followed by localization to the outer envelope due to a cleavable polyglycine region which serves as a “rejection signal” preventing translocation to the stroma (Inoue et al., 2001; Endow et al., 2016). TOC75-V was thought to lack a cleavable signal entirely, but the presence of a cleavable N-terminal signal has been confirmed (Gross et al., 2020). Despite earlier suggestions that outer envelope proteins insert spontaneously into the membrane (Jarvis and Robinson 2004), many still require a proteinaceous cofactor, likely TOC75 (Tu et al., 2004; Hofmann and Theg, 2005). In general one differentiates three classes of outer membrane proteins: signal-anchored (SA), tail-anchored (TA), and *ß*-barrel proteins (Fish et al., 2022).

SA anchored proteins feature a non-cleavable N-terminal moderately hydrophobic region, that will be inserted into the membrane, followed by a positively charged region to the C-terminal (Lee et al., 2011). However the mechanism of insertion for SA protein is less well understood apart from the involvement of cytosolic ankyrin repeat proteins Akr2A and Akr2B, which bind simultaneously to cytosolic ribosomes during translation and to lipids in the chloroplast outer membrane, thus decreasing the requirement for interaction with the GTPases (Dhanoa et al., 2010; Kim et al., 2014; 2015).

Similar to SA proteins, TA proteins feature a transmembrane domain near their C-terminus, however for at least some TA proteins, this is flanked by an RK/ST motif (Teresinski et al., 2019). As for the SA proteins, the pathway involves early ankyrin repeat protein binding. In a later stage, the guided entry of TA proteins and transmembrane recognition complex pathway proteins seem to be responsible for insertion (Formighieri et al., 2013). Finally, *ß*-barrel proteins which are also found in mitochondria and bacteria interact with the *ß*-barrel assembly machinery (Jores et al., 2016). *ß*-barrel proteins localized to the outer membrane still require the TOC complex to cross the outer membrane, and then TOC75-V to insert into the membrane (Gross et al., 2021).

Some inner envelope-localized proteins appear to bypass TIC-mediated translocation and are GTP-independent, likely requiring the TOC75 channel but bypassing the GTPase-mediated switch as they do not become imported to the stroma. The “stop-transfer” pathway uses a lateral insertion mechanism at TIC to insert directly without passing through a stromal intermediate stage, with known examples including albino or pale green mutant 1 (APG1) and accumulation and replication of chloroplasts 6 (ARC6) (Knight and Gray, 1995; Viana et al., 2010; Froehlich and Keegstra, 2011). The proposed mechanism is based on bulky hydrophobic residues of the mature transmembrane domains, but high glycine content and low proline content appear to also have a role (Froehlich and Keegstra, 2011). An alternative non-canonical route to the stop-transfer pathway involves PRAT proteins HP20, HP30, and HP30-2, which have been demonstrated to cooperate to mediate import of proteins without transit peptides such as TIC32 to the inner membrane of the chloroplast (Rossig et al., 2013; 2021), though the specific attributes that target proteins towards this translocon remain undetermined. More unusual examples of TIC-independent import include the soluble TIC22, which does not compete with stromal preprotein for translocation yet is still ATP-dependent and requires protease-sensitive proteins of the outer membrane (Kouranov et al., 1999).

In rare cases, chloroplast-targeted proteins that require glycosylation or other forms of specialized modification cannot use canonical import pathways. *a*-carbonic anhydrase 1 (CAH1) (Villarejo et al., 2005) and nucleotide pyrophosphatase/phosphodiesterase (NPP1) (Nanjo et al., 2006) use signal peptides to direct initial transport into the endoplasmic reticulum (ER), followed by TOC-independent import to chloroplasts. Vesicular fusion may deliver them to the intermembrane space, after which the glycosylated proteins could enter the stroma by vesicle budding from the outer membrane, through an unknown inner membrane transporter or by passage through the TIC translocon independent of TOC (Radhamony and Theg, 2006). Proteins inserted into the intermembrane space could bypass the TOC159 GTPase switch and engage with the TIC import machinery freely. While this method of protein import may appear conceptually simple and therefore seem like a more ancestral form of protein import, endomembrane system targeting is eukaryotic, not cyanobacterial in origin (Bodył et al., 2009; Gagat et al., 2013).

## 5 Characterization of the plastid proteome

A wealth of experimental data exists for chloroplast-targeted proteins in Arabidopsis, rice, and maize, represented in databases including AT_CHLORO (Ferro et al., 2010), Suba4 (Hooper et al., 2017), plprot (Kleffmann et al., 2006), and PPDB (Sun et al., 2009). Due to this exhaustive coverage, this review will not focus on chloroplast-targeted proteins and will instead examine plastid proteomics in non-green plastids and in non-model species. Understandably, such research has been hampered by the difficulty of isolating plastids from different types of non-green tissues. Notable studies published so far are summarized in Table 2. Due to the biological diversity of metabolic functions carried out by non-green plastids as well as significantly different isolation, detection, analysis, and curation methods, the capture of plastid proteome from a single development stage or tissue does not provide a comprehensive overview of plastid function. For instance, only 32% of the proteins identified in chromoplasts by Suzuki (Suzuki et al., 2015) overlapped with those identified in a previous proteomic analysis by Barsan (Barsan et al., 2010). This poses a major challenge in generalizing, as these non-green plastids are not static and are responding to environmental factors dynamically as green plastids and other organelles do.

**TABLE 2:** Summary of Non-Green Plastid Proteomics Studies. Characterization of non-green plastids are represented by only a handful of studies. However, several of the listed publications have found unique proteins in non-green morphotypes that are not present in green chloroplasts. Arabidopsis: *Arabidopsis thaliana*; Cauliflower: *Brassica oleracea*; sweet orange: *Citrus sinensis*; carrot: *Daucus carota*; kumquat: *Fortunella margarita;* medicago: *Medicago truncatula*; papaya: *Carica papaya*; pea: *Pisum sativum*; pepper: *Capsicum annuum*; rice: *Oryza sativa*; tobacco: *Nicotiana tabacum*; tomato: *Solanum lycopersicum*; watermelon: *Citrullus lanatus*; wheat: *Triticum aestivum*.

Publication	Species	Plastid type	Method	Number of unique plastid proteins	Overlap with chloroplast proteome
(Andon et al., 2002)	Wheat	Amyloplast	1-D/2-D gel, LC-MS/MS	171	N/A
(Baginsky et al., 2004)	Tobacco	Proplastid	(RP-LC)-MS/MS	168	121
			(reverse-phase LC); used both electrospray and nanospray ionization		
(von Zychlinski et al., 2005)	Rice	Etioplast	LC-NI MS/MS	216	N/A
(Ytterberg et al., 2006)	Arabidopsis	Chloroplast (Plastoglobuli)	nLC -MS/MS	32	N/A
			(Electrospray Ionization-tandem MS))		
(Siddique et al., 2006)	Pepper	Chromoplast	SDS-PAGE-(RP-LC)-MS/MS	151	N/A
(Balmer et al., 2006; Dupont 2008)	Wheat	Amyloplast	2-D gel, LC-MS/MS	180	N/A
(Kleffmann et al., 2006)	Rice	Etioplast, Chloroplast	2-D PAGE	477	N/A
(Kanervo et al., 2008)	Pea	Etioplast, Chloroplast	BN-PAGE, SDS-PAGE- > LC-ESI MS/MS	14	N/A
(Bräutigam and Weber 2009)	Cauliflower	Proplastid	MS/MS	226	N/A
(Barsan et al., 2010)	Tomato	Chromoplast	LC-MS/MS	988	577
(Daher et al., 2010)	Medicago	Nodular Leucoplasts	LC-MS/MS	266	N/A
(Zeng et al., 2011)	Sweet Orange	Chromoplast	SDS-PAGE-LC-MS/MS	418	N/A
(Barsan et al., 2012)	Tomato	Chloroplast, Chromoplast		1932	N/A
(Wang et al., 2013)	Tomato	Chromoplast	nLC-MS/MS	953	N/A
	Pepper			1752	
	Carrot			1891	
	Cauliflower			2262	
	Watermelon Papaya			1170	
				1581	
(Zeng et al., 2011)	Sweet Orange	Chromoplast	Titanium oxide affinity chromatography LC-MS/MS	109	N/A
(Suzuki et al., 2015)	Tomato	Chromoplast	GeLC-LC-MS/MS	605	82
(Daher et al., 2017)	Medicago	Nodular Leucoplasts	LC-MS/MS	490	N/A

Some generalizations can be made, however, based on experimental data. Commonly, chromoplasts are enriched in chlorophyll degradation, carotenoid storage, carotenoid synthesis, and jasmonic acid biosynthetic enzymes (Barsan et al., 2010; Zeng et al., 2011; Suzuki et al., 2015; Zhu et al., 2018; Rödiger et al., 2021). Elaioplasts of citrus peel are significantly more active in terpene synthesis compared to chromoplasts of the same tissue while having far fewer proteins involved in carotenoid metabolism (Zhu et al., 2018). Amyloplasts are most abundant in carbohydrate metabolism and hexose transporters as expected, but also contain significant lipid and amino acid biosynthesis proteins (Balmer et al., 2006; Dupont, 2008). Etioplasts contain much of the photosynthetic machinery with a few exceptions, as well as abundant amino acid and lipid biosynthesis enzymes (von Zychlinski et al., 2005; Kanervo et al., 2008). For all types of non-green plastids, enrichment of NTP translocators, hexose transporters, and carbohydrate metabolism enzymes point to heterotrophic but highly active metabolism. Similarly, abundant chaperone and heat shock proteins suggest that protein translation and import is extremely active in all plastid types, not just in chloroplasts. Finally, redox enzymes found in all plastids but especially abundant in chromoplasts allude to a need for pathogen defense, membrane protection, and reactive oxygen species detoxification. Up to 21 proteins involved in the ascorbate-glutathione cycle alone were found in tomato chromoplasts (Barsan et al., 2010). The xeroplast is a unique case of a non-green plastid which is not particularly metabolically active but acts as a survival structure depleted in many photosynthetic proteins and pigments, keeping biosynthetic building blocks stored in vesicles so chloroplast function can be reestablished upon rehydration (Ingle et al., 2008). The investigation of plastid proteomes beyond the chloroplast is critically needed to gain a holistic view of plastid proteomics and enable greater accuracy in predicting not only plastid localization but also categorization of different import classes (van Wijk and Baginsky, 2011).

Non-green plastids have also evolved outside of land plants, and even within single celled organisms, largely having proteomes which are reduced versions of the chloroplast proteome. Apicomplexa and Helicosporidium possess reduced plastids which engage in fatty acid synthesis, heme synthesis, carbohydrate metabolism, and amino acid synthesis, but have entirely lost their photosynthetic capacity (Lim and McFadden, 2010; Pombert et al., 2014; Boucher et al., 2018). The green alga *Polytomella parva* has non-photosynthetic plastids which lack a plastid genome and have a reduced TOC/TIC translocon of TOC33/34 and 75, and TIC 22, 40, and 110. These algal plastids retain a variety of biosynthetic processes including starch and sugar metabolism, amino acid synthesis, lipid metabolism, and redox homeostasis; the functions of a chloroplast are preserved with the exception of photosynthesis itself (Fuentes-Ramírez et al., 2021). Rhodelphis, a predatory red alga, has non-photosynthetic plastids which are mostly used in the production of heme and the assimilation of sulfur, perhaps the most reduced plastid mentioned thus far. These highly reduced plastids have experienced a loss of a plastid genome, and possess only a simple import apparatus of TOC75, TIC20, 22, and 32 (Gawryluk et al., 2019). For these single celled organisms, the development of non-green plastids represents a loss of unnecessary function, rather than a gain of new function as it does for many non-green plastid morphotypes in higher plants.

## 6 Algorithmic prediction of the plastid proteome

An pragmatic complement to experimental high-throughput proteomics is the use of computer algorithms to predict, annotate and compare plastid-targeted proteins. This methodology is limited to identifying localization without information regarding protein expression level or plastid morphotype. This method is relatively time- and cost-efficient compared to wet lab methods, and with proper application, can approach a higher level of accuracy. However, prediction software typically examines the N-terminal portion of protein models and uses either sequence and motif characteristics or annotation and sequence homology to determine localization. The lack of conserved sequence or domain structure in chloroplast transit peptides complicates prediction. TargetP (Emanuelsson et al., 2000; 2007; Juan et al., 2019), the most commonly-used program for predicting chloroplast-targeted proteins, performs with 46–86% sensitivity and 55–65% specificity when compared with curated mass spectrometry data (Zybailov et al., 2008; Christian et al., 2020b). Newer algorithms incorporating annotation and homology features as well as approaches using a combination of algorithms achieve even greater accuracy. In a comparison of Localizer, PredSL, TargetP, PCLR, MultiLoc2, and Wolf-PSORT using publicly-available organellar proteomics data, Localizer (Sperschneider et al., 2017) was found to be the single best program for plastid localization prediction with a MCC of 0.632. When 5 of these 6 programs were combined in each possible permutation (Wolf-PSORT was removed for poor performance), the best overall method was a “2 of 3” combination of TargetP, and Localizer with a MCC of 0.659 (Christian et al., 2020b). Combining predictive programs in this manner can utilize the strengths of each; for example, the high combined sensitivity and specificity led Localizer to be present in all of the top 25 combinations of programs, while Multiloc2’s unparalleled specificity enabled it to be present in many of the top combinations despite its poor sensitivity (Christian et al., 2020b).

Bioinformatics methods have largely been used on either small datasets or as a tool to curate mass spectrometry data, but several publications have applied them at the whole-genome level. The first such approach identified 2,261 proteins in Arabidopsis and 4,853 in rice (*Oryza sativa*) with predicted plastid localization; 880 and 817 of these proteins are thought to originate from the cyanobacteria (Richly and Leister, 2004). This study furthermore described that the number of non-essential genes outnumber essential genes and suggested that the majority of plastid-targeted proteins are eukaryotic in origin. This analysis was expanded to seven higher plant species, and the publication reported that only 737 proteins constituted the core, essential plastid-targeted genes (Schaeffer et al., 2014). Additionally, Schaeffer et al. reported a low of 795 species-specific plastid-targeted proteins in *Prunus persica* and a high of 4,817 in *Malus* × *domestica.* Arabidopsis alone had 2,154 species-specific plastid-targeted proteins. A more recent analysis of 15 plant genera representing a broad representation of Angiosperms found between 628 and 828 sequences to be shared among chloroplast proteomes of all species, and semi-conserved or species-specific plastid-targeted proteins were between six to 25 times more abundant (Christian et al., 2020a). Additionally, almost 1,000 gene loci in the Arabidopsis pan-genome have differential use of chloroplast transit peptides, and the same is true for nearly 9,000 gene families in the *Brachypodium distachyon* pan-genome (Christian et al., 2020b). Relatively few proteins are chloroplast-localized in all species, and most plastid-targeted proteins are likely to be taxa-specific or non-essential. However, not much is known about the function of these non-essential chloroplast genes, when they are expressed, or what plastid morphotype they accumulate in. Although this work is currently only predictive, the potential impact of non-essential plastid-targeted proteins merits further investigation to determine what metabolic roles different morphotypes play in specific tissues and in a species-specific manner.

Over the past decade machine learning has become increasingly commonplace, and the proliferation of machine learning tools and significant computational resources in the form of e.g. GPU computing easily accessible by non-experts has made it easier to develop effective predictive models from experimental data. Due to the highly diverse and difficult to understand nature of transit peptides and plastid import, as well as the increasing accessibility of machine learning tools, integrating proteomics studies with bioinformatic prediction is a promising avenue for expanding our understanding of plastid function. This is particularly true, if orthogonal non-sequence-based information is used to predict subcellular localization. Indeed, Ryngajllo et al. have shown in a proof of principle as early as 2011 that transcript expression contains information about plastidial localization that can be used for localization prediction (Ryngajllo et al., 2011). More recently MU-Loc has incorporated gene co-expression and other data into mitochondrial predictions to enable correct predictions for proteins lacking N-terminal pre-sequences (Zhang et al., 2018). Hence, such approaches might aid in the localization prediction of proteins imported by non-canonical pathways (see above), as typically -due to the lack of examples needed for training—only general signals are searched for. This approach of mixing data sources has been proven in studying the apicoplast, the non-photosynthetic plastid of apicomplexan protozoa. PlastNN was developed by applying a neural network to analyze the amino acid sequences of apicoplast targeted proteins and transcriptomic data from 8 time points; the result was an algorithm with a sensitivity and positive predictive value of 95% in predicting protein localization to the apicoplast, vastly outperforming prior algorithms (Boucher et al., 2018). The development of this model enabled the detection of several novel and essential proteins in an otherwise understudied plastid morphotype, using a relatively small dataset.

The accelerating rate at which proteomic data is generated through emerging methods like multiplexed proteomics (Pappireddi et al., 2019) only increases the value of machine learning tools. Machine learning is incredibly effective at detecting patterns, even incredibly complex ones when given an appropriately large amount of data; a phenomenon termed the “unreasonable effectiveness of data” (Halevy et al., 2009). While proteomic sampling may never be as cheap as words, the complexity of transit peptide sequence and chloroplast import seems to be a natural target for machine learning approaches. The decreasing time and resource cost of proteome analysis will enable increasingly accurate prediction of plastid protein import for a variety of plastid morphotypes and developmental stages, even in non-model species and the wide availability of such proteomic data sets allow to use semi-automated data mining approaches to produce training sets for machine learning approaches, that would otherwise have to be hand curated prior to training.

## 7 Conclusion

The evolutionary export of most of the plastid genome to the nuclear genome has left an exceptionally complex import system in its wake. Key aspects of protein import into the plastid still remain inadequately resolved as transit peptides remain enigmatic and difficult to predict while the translocons responsible for protein import remain a topic of debate. A universal model of protein translocation most likely does not exist, but the combination of modern high throughput proteomics and computing tools are making a headway in predicting and therefore understanding the characteristics and diversity of plastid transit peptides. The diverse metabolism, functionality and physiology of plastids across all plants makes them an ideal search ground for traits involved in environmental adaptation, alteration of photosynthetic efficiency, enhancement of nutrition and stress tolerance, and biosynthesis of novel bioactive compounds—information that is urgently needed to develop climate-resilient food crops and continue to feed the planet.

## References

[B1] Agne B. Infanger S. Wang F. Hofstetter V. Rahim G. Martin M. (2009). A Toc159 import receptor mutant, defective in hydrolysis of GTP, supports preprotein import into chloroplasts. J. Biol. Chem. 284, 8670–8679. 10.1074/jbc.M804235200 19188370 PMC2659226

[B2] Agne B. Kessler F. (2010). Modifications at the A-domain of the chloroplast import receptor Toc159. Planīt Signal Behav. 5, 1513–1516. 10.4161/psb.5.11.13707 PMC3115270 21057194

[B3] Ajjawi I. Lu Y. Savage L. J. Bell S. M. Last R. L. (2010). Large-scale reverse genetics in Arabidopsis: Case studies from the Chloroplast 2010 project. Plant Physiol. 152, 529–540. 10.1104/pp.109.148494 19906890 PMC2815874

[B4] Allen J. F. Raven J. A. (1996). Free-radical-induced mutation vs redox regulation: Costs and benefits of genes in organelles. J. Mol. Evol. 42, 482–492. 10.1007/BF02352278 8662000

[B5] Apel W. Schulze W. X. Bock R. (2010). Identification of protein stability determinants in chloroplasts. Plant J. 63, 636–650. 10.1111/j.1365-313X.2010.04268.x 20545891 PMC2988409

[B6] Armbruster U. Hertle A. Makarenko E. Zühlke J. Pribil M. Dietzmann A. (2009). Chloroplast proteins without cleavable transit peptides: Rare exceptions or a major constituent of the chloroplast proteome? Mol. Plant 2, 1325–1335. 10.1093/mp/ssp082 19995733

[B7] Aronsson H. Boij P. Patel R. Wardle A. Töpel M. Jarvis P. (2007). Toc64/OEP64 is not essential for the efficient import of proteins into chloroplasts in *Arabidopsis thaliana* . Plant J. 52, 53–68. 10.1111/j.1365-313X.2007.03207.x 17655652

[B8] Aronsson H. Combe J. Patel R. Jarvis P. (2006). *In vivo* assessment of the significance of phosphorylation of the Arabidopsis chloroplast protein import receptor, atToc33. FEBS Lett. 580, 649–655. 10.1016/j.febslet.2005.12.055 16412428

[B9] Bachmair A. Finley D. Varshavsky A. (1986). *In vivo* half-life of a protein is a function of its amino-terminal residue. Sci. (1979) 234, 179–186. 10.1126/science.3018930 3018930

[B10] Balmer Y. Vensel W. H. DuPont F. M. Buchanan B. B. Hurkman W. J. (2006). Proteome of amyloplasts isolated from developing wheat endosperm presents evidence of broad metabolic capability. J. Exp. Bot. 57, 1591–1602. 10.1093/jxb/erj156 16595579

[B11] Balsera M. Goetze T. A. Kovács-Bogdán E. Schürmann P. Wagner R. Buchanan B. B. (2009). Characterization of Tic110, a channel-forming protein at the inner envelope membrane of chloroplasts, unveils a response to Ca2+and a stromal regulatory disulfide bridge. J. Biol. Chem. 284, 2603–2616. 10.1074/jbc.M807134200 18986981

[B12] Balsera M. Soll J. Buchanan B. B. (2010). Redox extends its regulatory reach to chloroplast protein import. Trends Plant Sci. 15, 515–521. 10.1016/j.tplants.2010.06.002 20688558

[B13] Barsan C. Sanchez-bel P. Rombaldi C. Egea I. Rossignol M. (2010). Characteristics of the tomato chromoplast revealed by proteomic analysis. J. Exp. Bot. 61, 2413. 10.1093/jxb/erq070 20363867

[B14] Barsan C. Zouine M. Maza E. Bian W. Egea I. Rossignol M. (2012). Proteomic analysis of chloroplast-to-chromoplast transition in tomato reveals metabolic shifts coupled with disrupted thylakoid biogenesis machinery and elevated energy-production components. Plant Physiol. 160, 708–725. 10.1104/pp.112.203679 22908117 PMC3461550

[B15] Bartsch S. Monnet J. Selbach K. Quigley F. Gray J. Von Wettstein D. (2008). Three thioredoxin targets in the inner envelope membrane of chloroplasts function in protein import and chlorophyll metabolism. Proc. Natl. Acad. Sci. U. S. A. 105, 4933–4938. 10.1073/pnas.0800378105 18349143 PMC2290756

[B16] Bauer J. Chen K. Hiltbunner A. Wehrli E. Eugster M. Schnell D. (2000). The major protein import receptor of plastids is essential for chloroplast biogenesis. Nature 403, 203–207. 10.1038/35003214 10646606

[B17] Bell P. R. (1986). Features of egg cells of living representatives of ancient families of ferns. Ann. Bot. 57, 613–621. 10.1093/oxfordjournals.aob.a087144

[B18] Bodył A. Mackiewicz P. Stiller J. W. (2009). Early steps in plastid evolution: Current ideas and controversies. BioEssays 31, 1219–1232. 10.1002/bies.200900073 19847819

[B19] Boij P. Patel R. Garcia C. Jarvis P. Aronsson H. (2009). *In vivo* studies on the roles of Tic55-related proteins in chloroplast protein import in arabidopsis thaliana. Mol. Plant 2, 1397–1409. 10.1093/mp/ssp079 19995737

[B20] Bölter B. Soll J. (2016). Once upon a time – chloroplast protein import research from infancy to future challenges. Mol. Plant 9, 798–812. 10.1016/j.molp.2016.04.014 27142186

[B21] Bölter B. Soll J. (2017). Ycf1/Tic214 is not essential for the accumulation of plastid proteins. Mol. Plant 10, 219–221. 10.1016/j.molp.2016.10.012 27780781

[B22] Boucher M. J. Ghosh S. Zhang L. Lal A. Jang S. W. Ju A. (2018). Integrative proteomics and bioinformatic prediction enable a high-confidence apicoplast proteome in malaria parasites. PLoS Biol. 16, 20058955. 10.1371/journal.pbio.2005895 PMC6155542 30212465

[B23] Brillouet J. M. Romieu C. Schoefs B. Solymosi K. Cheynier V. Fulcrand H. (2013). The tannosome is an organelle forming condensed tannins in the chlorophyllous organs of Tracheophyta. Ann. Bot. 112, 1003–1014. 10.1093/aob/mct168 24026439 PMC3783233

[B24] Brillouet J. M. Verdeil J. L. Odoux E. Lartaud M. Grisoni M. Conéjéro G. (2014). Phenol homeostasis is ensured in vanilla fruit by storage under solid form in a new chloroplast-derived organelle, the phenyloplast. J. Exp. Bot. 65, 2427–2435. 10.1093/jxb/eru126 24683183 PMC4036510

[B25] Caliebe A. Grimm R. Kaiser G. Lübeck J. Soll J. Heins L. (1997). The chloroplastic protein import machinery contains a Rieske-type iron-sulfur cluster and a mononuclear iron-binding protein. EMBO J. 16, 7342–7350. 10.1093/emboj/16.24.7342 9405363 PMC1170334

[B26] Cao J. G. Dai X. F. Wang Q. X. (2012). Cytological features of oogenesis and their evolutionary significance in the fern Osmunda japonica. Sex. Plant Reprod. 25, 61–69. 10.1007/s00497-011-0179-7 22167247

[B27] Chen L. J. Li H. M. (2017). Stable megadalton TOC–TIC supercomplexes as major mediators of protein import into chloroplasts. Plant J. 92, 178–188. 10.1111/tpj.13643 28745032

[B28] Chen Y. L. Chen L. J. Chu C. C. Huang P. K. Wen J. R. Li H. min (2018). TIC236 links the outer and inner membrane translocons of the chloroplast. Nature 564, 125–129. 10.1038/s41586-018-0713-y 30464337

[B30] Chigri F. Hörmann F. Stamp A. Stammers D. K. Bölter B. Soll J. (2006). Calcium regulation of chloroplast protein translocation is mediated by calmodulin binding to Tic32. Proc. Natl. Acad. Sci. U. S. A. 103, 16051–16056. 10.1073/pnas.0607150103 17035502 PMC1635125

[B31] Chigri F. Soll J. Vothknecht U. C. (2005). Calcium regulation of chloroplast protein import. Plant J. 42, 821–831. 10.1111/j.1365-313X.2005.02414.x 15941396

[B32] Chotewutmontri P. Bruce B. D. (2015). Non-native, N-terminal Hsp70 molecular motor-recognition elements in transit peptides support plastid protein translocation. J. Biol. Chem. 290, 7602–7621. 10.1074/jbc.M114.633586 25645915 PMC4367265

[B33] Chotewutmontri P. Reddick L. E. Mcwilliams D. R. Campbell I. M. Bruce B. D. (2012). Differential transit peptide recognition during preprotein binding and translocation into flowering plant plastids. Plant Cell. 24, 3040–3059. 10.1105/tpc.112.098327 22829148 PMC3426131

[B34] Chou M. L. Liao W. Y. Wei W. C. Li A. Y. S. Chu C. Y. Wu C. L. (2018). The direct involvement of dark-induced Tic55 protein in chlorophyll catabolism and its indirect role in the MYB108-NAC signaling pathway during leaf senescence in *Arabidopsis thaliana* . Int. J. Mol. Sci. 19, 1854. 10.3390/ijms19071854 29937503 PMC6073118

[B35] Christian R. W. Hewitt S. L. Nelson G. Roalson E. H. Dhingra A. (2020a). Plastid transit peptides—where do they come from and where do they all belong? Multi-Genome and pan-genomic assessment of chloroplast transit peptide evolution. PeerJ 8, e9772. 10.7717/peerj.9772 32913678 PMC7456531

[B36] Christian R. W. Hewitt S. L. Roalson E. H. Dhingra A. (2020b). Genome-Scale characterization of predicted plastid-targeted proteomes in higher plants. Sci. Rep. 10, 8281. 10.1038/s41598-020-64670-5 32427841 PMC7237471

[B37] Chu C. C. Swamy K. Li H. M. (2020). Tissue-specific regulation of plastid protein import via transit-peptide motifs. Plant Cell. 32, 1204–1217. 10.1105/tpc.19.00702 32075863 PMC7145487

[B38] Chua N. H. Schmidt G. W. (1979). Transport of proteins into mitochondria and chloroplasts. J. Cell. Biol. 81, 461–483. 10.1083/jcb.81.3.461 379018 PMC2110399

[B39] Claros M. G. Brunak S. Von Heijne G. (1997). Prediction of N-terminal protein sorting signals. Curr. Opin. Struct. Biol. 7, 394–398. 10.1016/S0959-440X(97)80057-7 9204282

[B40] Cline K. Henry R. (1996). Import and routing of nucleus-encoded chloroplast proteins. Annu. Rev. Cell. Dev. Biol. 12, 1–26. 10.1146/annurev.cellbio.12.1.1 8970720

[B41] Coiffard C. Gomez B. Daviero-Gomez V. Dilcher D. L. (2012). Rise to dominance of angiosperm pioneers in European Cretaceous environments. Proc. Natl. Acad. Sci. 109, 20955–20959. 10.1073/pnas.1218633110 23213256 PMC3529080

[B42] de Boer A. D. Weisbeek P. J. Douwe de Boer A. (1991). Chloroplast protein topogenesis: Import, sorting and assembly. Biochimica Biophysica Acta - Rev. Biomembr. 1071, 221–253. 10.1016/0304-4157(91)90015-O 1958688

[B43] de Koning A. P. Keeling P. J. (2006). The complete plastid genome sequence of the parasitic green alga Helicosporidium sp. is highly reduced and structured. BMC Biol. 4, 12. 10.1186/1741-7007-4-12 16630350 PMC1463013

[B44] de Vries J. Sousa F. L. Bölter B. Soll J. Gould S. B. (2015). YCF1: A green tic? Plant Cell. 27, 1827–1833. 10.1105/tpc.114.135541 25818624 PMC4531346

[B45] Delaye L. Valadez-Cano C. Pérez-Zamorano B. (2016). How really ancient is Paulinella chromatophora? PLoS Curr. 8. 10.1371/currents.tol.e68a099364bb1a1e129a17b4e06b0c6b PMC4866557 28515968

[B46] Demarsy E. Lakshmanan A. M. Kessler F. (2014). Border control: Selectivity of chloroplast protein import and regulation at the TOC-complex. Front. Plant Sci. 5, 483. 10.3389/fpls.2014.00483 25278954 PMC4166117

[B47] Dhanoa P. K. Richardson L. G. L. Smith M. D. Gidda S. K. Henderson M. P. A. Andrews D. W. (2010). Distinct pathways mediate the sorting of tail-anchored proteins to the plastid outer envelope. PLoS One 5, e10098. 10.1371/journal.pone.0010098 20418952 PMC2854689

[B48] Dinh T. V. Bienvenut W. V. Linster E. Feldman-Salit A. Jung V. A. Meinnel T. (2015). Molecular identification and functional characterization of the first Nα-acetyltransferase in plastids by global acetylome profiling. Proteomics 15, 2426–2435. 10.1002/pmic.201500025 25951519 PMC4692087

[B49] Dong W. L. Jong K. K. Lee S. Choi S. Kim S. Hwang I. (2008). Arabidopsis nuclear-encoded plastid transit peptides contain multiple sequence subgroups with distinctive chloroplast-targeting sequence motifs. Plant Cell. 20, 1603–1622. 10.1105/tpc.108.060541 18552198 PMC2483360

[B50] Dupont F. M. (2008). Metabolic pathways of the wheat (*Triticum aestivum*) endosperm amyloplast revealed by proteomics. BMC Plant Biol. 8, 39–18. 10.1186/1471-2229-8-39 18419817 PMC2383896

[B51] Dutta S. Teresinski H. J. Smith M. D. (2014). A split-ubiquitin yeast two-hybrid screen to examine the substrate specificity of atToc159 and atToc132, two arabidopsis chloroplast preprotein import receptors. PLoS One 9, e95026. 10.1371/journal.pone.0095026 24736607 PMC3988174

[B52] Duy D. Wanner G. Meda A. R. von Wirén N. Soll J. Philippar K. (2007). PIC1, an ancient permease in Arabidopsis chloroplasts, mediates iron transport. Plant Cell. 19, 986–1006. 10.1105/tpc.106.047407 17337631 PMC1867359

[B53] Emanuelsson O. Brunak S. von Heijne G. Nielsen H. (2007). Locating proteins in the cell using TargetP, SignalP and related tools. Nat. Protoc. 2, 953–971. 10.1038/nprot.2007.131 17446895

[B54] Emanuelsson O. Nielsen H. Brunak S. von Heijne G. (2000). Predicting subcellular localization of proteins based on their N-terminal amino acid sequence. J. Mol. Biol. 300, 1005–1016. 10.1006/jmbi.2000.3903 10891285

[B55] Endow J. K. Rocha A. G. Baldwin A. J. Roston R. L. Yamaguchi T. Kamikubo H. (2016). Polyglycine acts as a rejection signal for protein transport at the chloroplast envelope. PLoS One 11, e0167802–e0167819. 10.1371/journal.pone.0167802 27936133 PMC5147994

[B56] Eseverri Á. Baysal C. Medina V. Capell T. Christou P. Rubio L. M. (2020). Transit peptides from photosynthesis-related proteins mediate import of a marker protein into different plastid types and within different species. Front. Plant Sci. 11, 560701–560714. 10.3389/fpls.2020.560701 33101328 PMC7545105

[B57] Falcón L. I. Magallón S. Castillo A. (2010). Dating the cyanobacterial ancestor of the chloroplast. ISME J. 4, 777–783. 10.1038/ismej.2010.2 20200567

[B58] Ferro M. Brugière S. Salvi D. Seigneurin-Berny D. Court M. Moyet L. (2010). AT_CHLORO, a comprehensive chloroplast proteome database with subplastidial localization and curated information on envelope proteins. Mol. Cell. Proteomics 9, 1063–1084. 10.1074/mcp.M900325-MCP200 20061580 PMC2877971

[B59] Figueroa-Martinez F. Jackson C. Reyes-Prieto A. (2019). Plastid genomes from diverse glaucophyte genera reveal a largely conserved gene content and limited architectural diversity. Genome Biol. Evol. 11, 174–188. 10.1093/gbe/evy268 30534986 PMC6330054

[B60] Fish M. Nash D. German A. Overton A. Jelokhani-Niaraki M. Chuong S. D. X. (2022). New insights into the chloroplast outer membrane proteome and associated targeting pathways. Int. J. Mol. Sci. 23, 1571. 10.3390/ijms23031571 35163495 PMC8836251

[B61] Formighieri C. Cazzaniga S. Kuras R. Bassi R. (2013). Biogenesis of photosynthetic complexes in the chloroplast of Chlamydomonas reinhardtii requires ARSA1, a homolog of prokaryotic arsenite transporter and eukaryotic TRC40 for guided entry of tail-anchored proteins. Plant J. 73, 850–861. 10.1111/tpj.12077 23167510

[B62] Froehlich J. E. Keegstra K. (2011). The role of the transmembrane domain in determining the targeting of membrane proteins to either the inner envelope or thylakoid membrane. Plant J. 68, 844–856. 10.1111/j.1365-313X.2011.04735.x 21838779

[B63] Fuentes-Ramírez E. O. Vázquez-Acevedo M. Cabrera-Orefice A. Guerrero-Castillo S. González-Halphen D. (2021). The plastid proteome of the nonphotosynthetic chlorophycean alga Polytomella parva. Microbiol. Res. 243, 126649. 10.1016/j.micres.2020.126649 33285428

[B64] Fulgosi H. Soll J. (2002). The chloroplast protein import receptors Toc34 and Toc159 are phosphorylated by distinct protein kinases. J. Biol. Chem. 277, 8934–8940. 10.1074/jbc.M110679200 11773075

[B65] Gabrielson P. W. Garbary D. J. Sommerfeld R. A. Townsend R. A. (1990). Rhodophyta. Burlington, MA, USA: Jones and Bartlett Publishers.

[B66] Gagat P. Bodył A. Mackiewicz P. (2013). How protein targeting to primary plastids via the endomembrane system could have evolved? A new hypothesis based on phylogenetic studies. Biol. Direct 8, 18. 10.1186/1745-6150-8-18 23845039 PMC3716720

[B67] Galpaz N. Wang Q. Menda N. Zamir D. Hirschberg J. (2008). Abscisic acid deficiency in the tomato mutant high-pigment 3 leading to increased plastid number and higher fruit lycopene content. Plant J. 53, 717–730. 10.1111/j.1365-313X.2007.03362.x 17988221

[B68] Ganesan I. Theg S. M. (2019). Structural considerations of folded protein import through the chloroplast TOC/TIC translocons. FEBS Lett. 593, 565–572. 10.1002/1873-3468.13342 30775779

[B69] Garrido C. Caspari O. D. Choquet Y. Wollman F. A. Lafontaine I. (2020). Evidence supporting an antimicrobial origin of targeting peptides to endosymbiotic organelles. Cells 9, 1795. 10.3390/cells9081795 32731621 PMC7463930

[B70] Gawryluk R. M. R. Tikhonenkov D. v. Hehenberger E. Husnik F. Mylnikov A. P. Keeling P. J. (2019). Non-photosynthetic predators are sister to red algae. Nature 572, 240–243. 10.1038/s41586-019-1398-6 31316212

[B71] Gould S. B. Waller R. F. McFadden G. I. (2008). Plastid evolution. Annu. Rev. Plant Biol. 59, 491–517. 10.1146/annurev.arplant.59.032607.092915 18315522

[B72] Green B. R. (2011). Chloroplast genomes of photosynthetic eukaryotes. Plant J. 66, 34–44. 10.1111/j.1365-313X.2011.04541.x 21443621

[B73] Gross L. E. Klinger A. Spies N. Ernst T. Flinner N. Simm S. (2021). Insertion of plastidic β-barrel proteins into the outer envelopes of plastids involves an intermembrane space intermediate formed with Toc75-V/OEP80. Plant Cell. 33, 1657–1681. 10.1093/plcell/koab052 33624803 PMC8254496

[B74] Gross L. E. Spies N. Simm S. Schleiff E. (2020). Toc75-V/OEP80 is processed during translocation into chloroplasts, and the membrane-embedded form exposes its POTRA domain to the intermembrane space. FEBS Open Bio 10, 444–454. 10.1002/2211-5463.12791 PMC7050246 31953987

[B75] Gutensohn M. Pahnke S. Kolukisaoglu Ü. Schulz B. Schierhorn A. Voigt A. (2004). Characterization of a T-DNA insertion mutant for the protein import receptor atToc33 from chloroplasts. Mol. Genet. Genomics 272, 379–396. 10.1007/s00438-004-1068-7 15517392

[B76] Gutensohn M. Schulz B. Nicolay P. Flügge U. I. (2000). Functional analysis of the two Arabidopsis homologues of Toc34, a component of the chloroplast protein import apparatus. Plant J. 23, 771–783. 10.1046/j.1365-313X.2000.00849.x 10998188

[B77] Halevy A. Norvig P. Pereira F. (2009). The unreasonable effectiveness of data. IEEE Intell. Syst. 24, 8–12. 10.1109/MIS.2009.36

[B78] Hauenstein M. Christ B. Das A. Aubry S. Hörtensteiner S. (2016). A role for TIC55 as a hydroxylase of phyllobilins, the products of chlorophyll breakdown during plant senescence. Plant Cell. 28, 2510–2527. 10.1105/tpc.16.00630 27655840 PMC5134989

[B79] Heins L. Mehrle A. Hemmler R. Wagner R. Küchler M. Hörmann F. (2002). The preprotein conducting channel at the inner envelope membrane of plastids. EMBO J. 21, 2616–2625. 10.1093/emboj/21.11.2616 12032074 PMC126020

[B80] Hiltbrunner A. Bauer J. Vidi P. A. Infanger S. Weibel P. Hohwy M. (2001). Targeting of an abundant cytosolic form of the protein import receptor at Toc159 to the outer chloroplast membrane. J. Cell. Biol. 154, 309–316. 10.1083/jcb.200104022 11470820 PMC2150772

[B82] Hirohashi T. Nakai M. (2000). Molecular cloning and characterization of maize Toc34, a regulatory component of the protein import machinery of chloroplast. Biochimica Biophysica Acta - Gene Struct. Expr. 1491, 309–314. 10.1016/S0167-4781(00)00043-9 10760596

[B83] Hofmann N. R. Theg S. M. (2005). Protein- and energy-mediated targeting of chloroplast outer envelope membrane proteins. Plant J. 44, 917–927. 10.1111/j.1365-313X.2005.02571.x 16359385

[B84] Holbrook K. Subramanian C. Chotewutmontri P. Reddick L. E. Wright S. Zhang H. (2016). Functional analysis of semi-conserved transit peptide motifs and mechanistic implications in precursor targeting and recognition. Mol. Plant 9, 1286–1301. 10.1016/j.molp.2016.06.004 27378725

[B85] Hooper C. M. Castleden I. R. Tanz S. K. Aryamanesh N. Millar A. H. (2017). SUBA4: The interactive data analysis centre for Arabidopsis subcellular protein locations. Nucleic Acids Res. 45, D1064–D1074. 10.1093/nar/gkw1041 27899614 PMC5210537

[B86] Hörmann F. Küchler M. Sveshnikov D. Oppermann U. Li Y. Soll J. (2004). Tic32, an essential component in chloroplast biogenesis. J. Biol. Chem. 279, 34756–34762. 10.1074/jbc.M402817200 15180984

[B87] Howe C. J. Barbrook A. C. Nisbet R. E. R. Lockhart P. J. Larkum A. W. D. (2008). The origin of plastids. Philos. Trans. R. Soc. Lond B Biol. Sci. 363, 2675–2685. 10.1098/rstb.2008.0050 18468982 PMC2606771

[B88] Huang C. Y. Ayliffe M. A. Timmis J. N. (2003). Direct measurement of the transfer rate of chloroplast DNA into the nucleus. Nature 422, 72–76. 10.1038/nature01435 12594458

[B89] Huang P. K. Chan P. T. Su P. H. Chen L. J. Li H. (2016). Chloroplast Hsp93 directly binds to transit peptides at an early stage of the preprotein import process. Plant Physiol. 170, 857–866. 10.1104/pp.15.01830 26676256 PMC4734592

[B90] Inaba T. Schnell D. J. (2008). Protein trafficking to plastids: One theme, many variations. Biochem. J. 413, 15–28. 10.1042/BJ20080490 18537794

[B91] Ingle R. A. Collett H. Cooper K. Takahashi Y. Farrant J. M. Illing N. (2008). Chloroplast biogenesis during rehydration of the resurrection plant xerophyta humilis: Parallels to the etioplast-chloroplast transition. Plant Cell. Environ. 31, 1813–1824. 10.1111/j.1365-3040.2008.01887.x 18771571

[B92] Inoue H. Li M. Schnell D. J. (2013). An essential role for chloroplast heat shock protein 90 (Hsp90C) in protein import into chloroplasts. Proc. Natl. Acad. Sci. U. S. A. 110, 3173–3178. 10.1073/pnas.1219229110 23382192 PMC3581895

[B93] Inoue H. Rounds C. Schnell D. J. (2010). The molecular basis for distinct pathways for protein import into arabidopsis chloroplasts. Plant Cell. 22, 1947–1960. 10.1105/tpc.110.074328 20562235 PMC2910967

[B94] Inoue K. Demel R. De Kruijff B. Keegstra K. (2001). The N-terminal portion of the preToc75 transit peptide interacts with membrane lipids and inhibits binding and import of precursor proteins into isolated chloroplasts. Eur. J. Biochem. 268, 4036–4043. 10.1046/j.1432-1327.2001.02316.x 11453998

[B95] Inoue K. Potter D. (2004). The chloroplastic protein translocation channel Toc75 and its paralog OEP80 represent two distinct protein families and are targeted to the chloroplastic outer envelope by different mechanisms. Plant J. 39, 354–365. 10.1111/j.1365-313X.2004.02135.x 15255865

[B96] Ivanova Y. Smith M. D. Chen K. Schnell D. J. (2004). Members of the Toc159 import receptor family represent distinct pathways for protein targeting to plastids. Mol. Biol. Cell. 15, 3379–3392. 10.1091/mbc.e03-12-0923 15090618 PMC452591

[B97] Jackson-Constan D. Keegstra K. (2001). Arabidopsis genes encoding components of the chloroplastic protein import apparatus. Plant Physiol. 125, 1567–1576. 10.1104/pp.125.4.1567 11299338 PMC88814

[B98] Jarvis P. Chen L. J. Li H. Peto C. A. Fankhauser C. Chory J. (1998). An Arabidopsis mutant defective in the plastid general protein import apparatus. Science 282, 100–103. 10.1126/science.282.5386.100 9756470

[B99] Jelic M. Soll J. Schleiff E. (2003). Two Toc34 homologues with different properties. Biochemistry 42, 5906–5916. 10.1021/bi034001q 12741849

[B100] Jelic M. Sveshnikova N. Motzkus M. Hörth P. Soll J. Schleiff E. (2002). The chloroplast import receptor Toc34 functions as preprotein-regulated GTPase. Biol. Chem. 383, 1875–1883. 10.1515/BC.2002.211 12553724

[B101] Jiao Y. Wickett N. J. Ayyampalayam S. Chanderbali A. S. Landherr L. Ralph P. E. (2011). Ancestral polyploidy in seed plants and angiosperms. Nature 473, 97–100. 10.1038/nature09916 21478875

[B102] Jin Z. Wan L. Zhang Y. Li X. Cao Y. Liu H. (2022). Structure of a TOC-TIC supercomplex spanning two chloroplast envelope membranes. Cell. 185, 4788–4800.e13. 10.1016/j.cell.2022.10.030 36413996

[B103] Jores T. Klinger A. Groß L. E. Kawano S. Flinner N. Duchardt-Ferner E. (2016). Characterization of the targeting signal in mitochondrial β-barrel proteins. Nat. Commun. 7, 12036. 10.1038/ncomms12036 27345737 PMC4931251

[B104] Juan J. Armenteros A. Salvatore M. Emanuelsson O. Winther O. Von Heijne G. (2019). Detecting sequence signals in targeting peptides using deep learning. Life Sci. Alliance 2, 2019004299. 10.26508/lsa.201900429 PMC6769257 31570514

[B105] Kaneko T. Sato S. Kotani H. Tanaka A. Asamizu E. Nakamura Y. (1996). Sequence analysis of the genome of the unicellular cyanobacterium synechocystis sp. strain PCC6803. II. Sequence determination of the entire genome and assignment of potential protein-coding regions. DNA Res. 3, 109–136. 10.1093/dnares/3.3.109 8905231

[B106] Kanervo E. Singh M. Suorsa M. Paakkarinen V. Aro E. Battchikova N. (2008). Expression of protein complexes and individual proteins upon transition of etioplasts to chloroplasts in pea (Pisum sativum). Plant Cell. Physiol. 49, 396–410. 10.1093/pcp/pcn016 18263621

[B107] Karlin-Neumann G. A. Tobin E. M. (1986). Transit peptides of nuclear-encoded chloroplast proteins share a common amino acid framework. EMBO J. 5, 9–13. 10.1002/j.1460-2075.1986.tb04170.x 3514209 PMC1166688

[B108] Kasmati A. R. Töpel M. Patel R. Murtaza G. Jarvis P. (2011). Molecular and genetic analyses of Tic20 homologues in *Arabidopsis thaliana* chloroplasts. Plant J. 66, 877–889. 10.1111/j.1365-313X.2011.04551.x 21395885

[B109] Keeling P. J. (2010). The endosymbiotic origin, diversification and fate of plastids. Philosophical Trans. R. Soc. B Biol. Sci. 365, 729–748. 10.1098/rstb.2009.0103 PMC2817223 20124341

[B110] Kessler F. Blobel G. Patel H. A. Schnell D. J. (1994). Identification of two GTP-binding proteins in the chloroplast protein import machinery. Science 266, 1035–1039. 10.1126/science.7973656 7973656

[B111] Kessler F. Schnell D. (2009). Chloroplast biogenesis: Diversity and regulation of the protein import apparatus. Curr. Opin. Cell. Biol. 21, 494–500. 10.1016/j.ceb.2009.03.004 19410443

[B112] Kessler F. Schnell D. J. (2006). The function and diversity of plastid protein import pathways: A multilane GTPase highway into plastids. Traffic 7, 248–257. 10.1111/j.1600-0854.2005.00382.x 16497220

[B113] Kikuchi S. Asakura Y. Imai M. Nakahira Y. Kotani Y. Hashiguchi Y. (2018). A ycf2-FtsHi heteromeric AAA-ATPase complex is required for chloroplast protein import. Plant Cell. 30, 2677–2703. 10.1105/tpc.18.00357 30309901 PMC6305978

[B114] Kikuchi S. Bédard J. Hirano M. Hirabayashi Y. Oishi M. Imai M. (2013). Uncovering the protein translocon at the chloroplast inner envelope membrane. Sci. (1979) 339, 571–574. 10.1126/science.1229262 23372012

[B115] Kikuchi S. Oishi M. Hirabayashi Y. Lee D. W. Hwang I. Nakai M. (2009). A 1 -Megadalton translocation complex containing tic20 and tic21 mediates chloroplast protein import at the inner envelope membrane. Plant Cell. 21, 1781–1797. 10.1105/tpc.108.063552 19531596 PMC2714928

[B116] Kim D. H. Lee J. E. Xu Z. Y. Geem K. R. Kwon Y. Park J. W. (2015). Cytosolic targeting factor AKR2A captures chloroplast outer membrane-localized client proteins at the ribosome during translation. Nat. Commun. 6, 6843. 10.1038/ncomms7843 25880450

[B117] Kim D. H. Park M. J. Gwon G. H. Silkov A. Xu Z. Y. Yang E. C. (2014). An ankyrin repeat domain of AKR2 drives chloroplast targeting through coincident binding of two chloroplast lipids. Dev. Cell. 30, 598–609. 10.1016/j.devcel.2014.07.026 25203210 PMC4170656

[B118] Kleffmann T. Hirsch-Hoffmann M. Gruissem W. Baginsky S. (2006). plprot: A comprehensive proteome database for different plastid types. Plant Cell. Physiol. 47, 432–436. 10.1093/pcp/pcj005 16418230

[B119] Knight J. S. Gray J. C. (1995). The N-terminal hydrophobic region of the mature phosphate translocator is sufficient for targeting to the chloroplast inner envelope membrane. Plant Cell. 7, 1421–1432. 10.1105/tpc.7.9.1421 8589626 PMC160965

[B120] Koenig P. Oreb M. Höfle A. Kaltofen S. Rippe K. Sinning I. (2008). The GTPase cycle of the chloroplast import receptors toc33/toc34: Implications from monomeric and dimeric structures. Structure 16, 585–596. 10.1016/j.str.2008.01.008 18400179

[B121] Köhler D. Montandon C. Hause G. Majovsky P. Kessler F. Baginsky S. (2015). Characterization of chloroplast protein import without Tic56, a component of the 1-megadalton translocon at the inner envelope membrane of chloroplasts. Plant Physiol. 167, 972–990. 10.1104/pp.114.255562 25588737 PMC4348784

[B122] Kouranov A. Chen X. Fuks B. Schnell D. J. (1998). Tic20 and Tic22 are new components of the protein import apparatus at the chloroplast inner envelope membrane. J. Cell. Biol. 143, 991–1002. 10.1083/jcb.143.4.991 9817756 PMC2132967

[B123] Kouranov A. Wang H. Schnell D. J. (1999). Tic22 is targeted to the intermembrane space of chloroplasts by a novel pathway. J. Biol. Chem. 274, 25181–25186. 10.1074/jbc.274.35.25181 10455201

[B124] Kovács-Bogdán E. Benz J. P. Soll J. Bölter B. (2011). Tic20 forms a channel independent of Tic110 in chloroplasts. BMC Plant Biol. 11, 133. 10.1186/1471-2229-11-133 21961525 PMC3203047

[B125] Kubis S. Patel R. Combe J. Bédard J. Kovacheva S. Lilley K. (2004). Functional specialization amongst the Arabidopsis Toc159 family of chloroplast protein import receptors. Plant Cell. 16, 2059–2077. 10.1105/tpc.104.023309 15273297 PMC519198

[B126] Küchler M. Dicker S. Hörmann F. Soll J. Heins L. (2002). Protein import into chloroplasts involves redox-regulated proteins. Curr. Opin. Plant Biol. 21, 6136–6145. 10.1093/emboj/cdf621 PMC137210 12426385

[B127] Lamberti G. Drurey C. Soll J. Schwenkert S. (2011a). The phosphorylation state of chloroplast transit peptides regulates preprotein import. Plant Signal Behav. 6, 1918–1920. 10.4161/psb.6.12.18127 22105029 PMC3337178

[B128] Lamberti G. Gügel I. L. Meurer J. Soll J. Schwenkert S. (2011b). The cytosolic kinases STY8, STY17, and STY46 are involved in chloroplast differentiation in arabidopsis. Plant Physiol. 157, 70–85. 10.1104/pp.111.182774 21799034 PMC3165899

[B129] Lee D. W. Lee J. Hwang I. (2017). Sorting of nuclear-encoded chloroplast membrane proteins. Curr. Opin. Plant Biol. 40, 1–7. 10.1016/j.pbi.2017.06.011 28668581

[B130] Lee D. W. Lee S. Lee J. Woo S. Razzak M. A. Vitale A. (2019). Molecular mechanism of the specificity of protein import into chloroplasts and mitochondria in plant cells. Mol. Plant 12, 951–966. 10.1016/j.molp.2019.03.003 30890495

[B131] Lee D. W. Woo S. Geem K. R. Hwang I. (2015). Sequence motifs in transit peptides act as independent functional units and can be transferred to new sequence contexts. Plant Physiol. 169, 471–484. 10.1104/pp.15.00842 26149569 PMC4577419

[B132] Lee J. Lee H. Kim J. Lee S. Kim D. H. Kim S. (2011). Both the hydrophobicity and a positively charged region flanking the C-terminal region of the transmembrane domain of signal-anchored proteins play critical roles in determining their targeting specificity to the endoplasmic reticulum or endosymbiotic organelles in Arabidopsis cells. Plant Cell. 23, 1588–1607. 10.1105/tpc.110.082230 21515817 PMC3101543

[B133] Li H. M. Chen L. J. (1997). A novel chloroplastic outer membrane-targeting signal that functions at both termini of passenger polypeptides. J. Biol. Chem. 272, 10968–10974. 10.1074/jbc.272.16.10968 9099756

[B134] Li H. M. Chiu C. C. (2010). Protein transport into chloroplasts. Annu. Rev. Plant Biol. 61, 157–180. 10.1146/annurev-arplant-042809-112222 20192748

[B135] Li H. M. Schnell D. Theg S. M. (2020). Protein import motors in chloroplasts: On the role of chaperones. Plant Cell. 32, 536–542. 10.1105/tpc.19.00300 31932485 PMC7054032

[B136] Li H. M. Teng Y. S. (2013). Transit peptide design and plastid import regulation. Trends Plant Sci. 18, 360–366. 10.1016/j.tplants.2013.04.003 23688728

[B137] Liebers M. Grübler B. Chevalier F. Lerbs-Mache S. Merendino L. Blanvillain R. (2017). Regulatory shifts in plastid transcription play a key role in morphological conversions of plastids during plant development. Front. Plant Sci. 8, 23. 10.3389/fpls.2017.00023 28154576 PMC5243808

[B138] Lim L. McFadden G. I. (2010). The evolution, metabolism and functions of the apicoplast. Philosophical Trans. R. Soc. B Biol. Sci. 365, 749–763. 10.1098/rstb.2009.0273 PMC2817234 20124342

[B139] Ling Q. Broad W. Trösch R. Töpel M. Demiral Sert T. Lymperopoulos P. (2019). Ubiquitin-dependent chloroplast-associated protein degradation in plants. Sci. (1979) 363, eaav4467. 10.1126/science.aav4467 30792274

[B140] Ling Q. Huang W. Baldwin A. Jarvis P. (2012). Chloroplast biogenesis is regulated by direct action of the ubiquitin-proteasome system. Science 338, 655–659. 10.1126/science.1225053 23118188

[B141] Ling Q. Jarvis P. (2015). Regulation of chloroplast protein import by the ubiquitin E3 ligase SP1 is important for stress tolerance in plants. Curr. Biol. 25, 2527–2534. 10.1016/j.cub.2015.08.015 26387714 PMC4598742

[B142] Ling Q. Sadali N. M. Soufi Z. Zhou Y. Huang B. Zeng Y. (2021). The chloroplast-associated protein degradation pathway controls chromoplast development and fruit ripening in tomato. Nat. Plants 7, 655–666. 10.1038/s41477-021-00916-y 34007040

[B143] Liu L. McNeilage R. T. Shi L. X. Theg S. M. (2014). ATP requirement for chloroplast protein import is set by the Km for ATP hydrolysis of stromal Hsp70 in Physcomitrella patens. Plant Cell. 26, 1246–1255. 10.1105/tpc.113.121822 24596240 PMC4001381

[B144] Loudya N. Maffei D. P. F. Bdard J. Ali S. M. Devlin P. F. Paul Jarvis R. (2022). Mutations in the chloroplast inner envelope protein TIC100 impair and repair chloroplast protein import and impact retrograde signaling. Plant Cell. 34, 3028–3046. 10.1093/plcell/koac153 35640571 PMC9338805

[B145] Lynch M. Blanchard J. L. (1998). Deleterious mutation accumulation in organelle genomes. Genetica 102–103, 29–39. 10.1007/978-94-011-5210-5_3 9720269

[B146] Mackenzie S. A. (2005). Plant organellar protein targeting: A traffic plan still under construction. Trends Cell. Biol. 15, 548–554. 10.1016/j.tcb.2005.08.007 16143534

[B147] Maréchal E. Cesbron-Delauw M. F. (2001). The apicoplast: A new member of the plastid family. Trends Plant Sci. 6, 200–205. 10.1016/S1360-1385(01)01921-5 11335172

[B148] Martin W. Herrmann R. G. (1998). Gene transfer from organelles to the nucleus: How much, what happens, and why? Plant Physiol. 118, 9–17. 10.1104/pp.118.1.9 9733521 PMC1539188

[B149] Martin W. Rujan T. Richly E. Hansen A. Cornelsen S. Lins T. (2002). Evolutionary analysis of Arabidopsis, cyanobacterial, and chloroplast genomes reveals plastid phylogeny and thousands of cyanobacterial genes in the nucleus. Proc. Natl. Acad. Sci. U. S. A. 99, 12246–12251. 10.1073/pnas.182432999 12218172 PMC129430

[B150] May T. Soll J. (2000). 14-3-3 proteins form a guidance complex with chloroplast precursor proteins in plants. Plant Cell. 12, 53–64. 10.1105/tpc.12.1.53 10634907 PMC140214

[B151] McFadden G. I. (1999). Endosymbiosis and evolution of the plant cell. Curr. Opin. Plant Biol. 2, 513–519. 10.1016/S1369-5266(99)00025-4 10607659

[B152] McFadden G. I. Van Dooren G. G. (2004). Evolution: Red algal genome affirms a common origin of all plastids. Curr. Biol. 14, 514–516. 10.1016/j.cub.2004.06.041 15242632

[B153] Meeks J. C. Elhai J. Thiel T. Potts M. Larimer F. Lamerdin J. (2001). An overview of the genome of Nostoc punctiforme, a multicellular, symbiotic cyanobacterium. Photosynth Res. 70, 85–106. 10.1023/A:1013840025518 16228364

[B154] Muller H. J. (1964). The relation of recombination to mutational advance. Mutat. Res. 1, 2–9. 10.1016/0027-5107(64)90047-8 14195748

[B155] Muñoz-gómez S. A. Kreutz M. Hess S. (2021). A microbial eukaryote with a unique combination of purple bacteria and green algae as endosymbionts. Sci. Adv. 7, 1–12. 10.1126/sciadv.abg4102 PMC8195481 34117067

[B156] Muraki N. Nomata J. Ebata K. Mizoguchi T. Shiba T. Tamiaki H. (2010). X-ray crystal structure of the light-independent protochlorophyllide reductase. Nature 465, 110–114. 10.1038/nature08950 20400946

[B157] Nakai M. (2015a). The TIC complex uncovered: The alternative view on the molecular mechanism of protein translocation across the inner envelope membrane of chloroplasts. Biochim. Biophys. Acta 1847, 957–967. 10.1016/j.bbabio.2015.02.011 25689609

[B158] Nakai M. (2015b). YCF1: A green tic: Response to the de Vries et al. Commentary. Plant Cell. 27, 1834–1838. 10.1105/tpc.15.00363 26071422 PMC4531358

[B159] Nakrieko K. A. Mould R. M. Smith A. G. (2004). Fidelity of targeting to chloroplasts is not affected by removal of the phosphorylation site from the transit peptide. Eur. J. Biochem. 271, 509–516. 10.1046/j.1432-1033.2003.03950.x 14728677

[B160] Nanjo Y. Oka H. Ikarashi N. Kaneko K. Kitajima A. Mitsui T. (2006). Rice plastidial N-glycosylated nucleotide pyrophosphatase/phosphodiesterase is transported from the ER-golgi to the chloroplast through the secretory pathway. Plant Cell. 18, 2582–2592. 10.1105/tpc.105.039891 17028208 PMC1626603

[B161] Nicolay K. Laterveer F. D. van Heerde W. L. (1994). Effects of amphipathic peptides, including presequences, on the functional integrity of rat liver mitochondrial membranes. J. Bioenerg. Biomembr. 26, 327–334. 10.1007/BF00763104 8077186

[B162] Nowack E. C. M. Melkonian M. Glöckner G. (2008). Chromatophore genome sequence of Paulinella sheds light on acquisition of photosynthesis by eukaryotes. Curr. Biol. 18, 410–418. 10.1016/j.cub.2008.02.051 18356055

[B163] Nowack E. C. M. Vogel H. Groth M. Grossman A. R. Melkonian M. Glöckner G. (2011). Endosymbiotic gene transfer and transcriptional regulation of transferred genes in Paulinella chromatophora. Mol. Biol. Evol. 28, 407–422. 10.1093/molbev/msq209 20702568

[B166] Pappireddi N. Martin L. Wühr M. (2019). A review on quantitative multiplexed proteomics. Chembiochem 20, 1210–1224. 10.1002/cbic.201800650 30609196 PMC6520187

[B167] Patron N. J. Waller R. F. (2007). Transit peptide diversity and divergence: A global analysis of plastid targeting signals. BioEssays 29, 1048–1058. 10.1002/bies.20638 17876808

[B168] Pilon M. Wienk H. Sips W. De Swaaf M. Talboom I. Van’t Hof R. (1995). Functional domains of the ferredoxin transit sequence involved in chloroplast import. J. Biol. Chem. 270, 3882–3893. 10.1074/jbc.270.8.3882 7876133

[B169] Pinnaduwage P. Bruce B. D. (1996). *In vitro* interaction between a chloroplast transit peptide and chloroplast outer envelope lipids is sequence-specific and lipid class-dependent. J. Biol. Chem. 271, 32907–32915. 10.1074/jbc.271.51.32907 8955132

[B170] Pombert J. F. Blouin N. A. Lane C. Boucias D. Keeling P. J. (2014). A lack of parasitic reduction in the obligate parasitic green alga Helicosporidium. PLoS Genet. 10, e1004355. 10.1371/journal.pgen.1004355 24809511 PMC4014436

[B171] Qbadou S. Becker T. Mirus O. Tews I. Soll J. Schleiff E. (2006). The molecular chaperone Hsp90 delivers precursor proteins to the chloroplast import receptor Toc64. EMBO J. 25, 1836–1847. 10.1038/sj.emboj.7601091 16619024 PMC1456943

[B172] Qiu H. Price D. C. Weber A. P. M. Facchinelli F. Yoon H. S. Bhattacharya D. (2013). Assessing the bacterial contribution to the plastid proteome. Trends Plant Sci. 18, 680–687. 10.1016/j.tplants.2013.09.007 24139901

[B173] Radhamony R. N. Theg S. M. (2006). Evidence for an ER to Golgi to chloroplast protein transport pathway. Trends Cell. Biol. 16, 385–387. 10.1016/j.tcb.2006.06.003 16815014

[B174] Reddick L. E. Vaughn M. D. Wright S. J. Campbell I. M. Bruce B. D. (2007). *In vitro* comparative kinetic analysis of the chloroplast Toc GTPases. J. Biol. Chem. 282, 11410–11426. 10.1074/jbc.M609491200 17261588

[B175] Reiland S. Grossmann J. Baerenfaller K. Gehrig P. Nunes-Nesi A. Fernie A. R. (2011). Integrated proteome and metabolite analysis of the de-etiolation process in plastids from rice (Oryza sativa L.). Proteomics 11, 1751–1763. 10.1002/pmic.201000703 21433289

[B176] Reumann S. Davila-Aponte J. Keegstra K. (1999). The evolutionary origin of the protein-translocating channel of chloroplastic envelope membranes: Identification of a cyanobacterial homolog. Proc. Natl. Acad. Sci. 96, 784–789. 10.1073/pnas.96.2.784 9892711 PMC15214

[B177] Reumann S. Keegstra K. (1999). The endosymbiotic origin of the protein import machinery of chloroplastic envelope membranes. Trends Plant Sci. 4, 302–307. 10.1016/S1360-1385(99)01449-1 10431219

[B178] Richardson L. G. Small E. L. Inoue H. Schnell D. J. (2018). Molecular topology of the transit peptide during chloroplast protein import. Plant Cell. 30, 1789–1806. 10.1105/tpc.18.00172 29991536 PMC6139696

[B179] Richly E. Leister D. (2004). An improved prediction of chloroplast proteins reveals diversities and commonalities in the chloroplast proteomes of Arabidopsis and rice. Gene 329, 11–16. 10.1016/j.gene.2004.01.008 15033524

[B180] Richter S. Lamppa G. K. (1998). A chloroplast processing enzyme functions as the general stromal processing peptidase. Proc. Natl. Acad. Sci. U. S. A. 95, 7463–7468. 10.1073/pnas.95.13.7463 9636172 PMC22651

[B181] Richter S. Lamppa G. K. (1999). Stromal processing peptidase binds transit peptides and initiates their atp-dependent turnover in chloroplasts. J. Cell. Biol. 147, 33–43. 10.1083/jcb.147.1.33 10508853 PMC2164977

[B182] Rödiger A. Agne B. Dobritzsch D. Helm S. Müller F. Pötzsch N. (2021). Chromoplast differentiation in bell pepper (Capsicum annuum) fruits. Plant J. 105, 1431–1442. 10.1111/tpj.15104 33258209

[B183] Rolland V. Badger M. R. Price G. D. (2016). Redirecting the cyanobacterial bicarbonate transporters BicA and SbtA to the chloroplast envelope: Soluble and membrane cargos need different chloroplast targeting signals in plants. Front. Plant Sci. 7, 185. 10.3389/fpls.2016.00185 26973659 PMC4770052

[B184] Rossig C. Gray J. Valdes O. Springer A. Rustgi S. von Wettstein D. (2021). Prat proteins operate in organellar protein import and export in arabidopsis thaliana. Plants 10, 958. 10.3390/plants10050958 34064964 PMC8151980

[B185] Rossig C. Reinbothe C. Gray J. Valdes O. Von Wettstein D. Reinbothe S. (2013). Three proteins mediate import of transit sequence-less precursors into the inner envelope of chloroplasts in arabidopsis thaliana. Proc. Natl. Acad. Sci. U. S. A. 110, 19962–19967. 10.1073/pnas.1319648110 24248378 PMC3856848

[B186] Row P. E. Gray J. C. (2001). Chloroplast precursor proteins compete to form early import intermediates in isolated pea chloroplasts. J. Exp. Bot. 52, 47–56. 10.1093/jexbot/52.354.47 11181712

[B187] Rowland E. Kim J. Bhuiyan N. H. van Wijk K. J. (2015). The arabidopsis chloroplast stromal N-terminome: Complexities of amino-terminal protein maturation and stability. Plant Physiol. 169, 1881–1896. 10.1104/pp.15.01214 26371235 PMC4634096

[B188] Ryngajllo M. Childs L. Lohse M. Giorgi F. M. Lude A. Selbig J. (2011). SLocX: Predicting subcellular localization of arabidopsis proteins leveraging gene expression data. Front. Plant Sci. 2, 43. 10.3389/fpls.2011.00043 22639594 PMC3355584

[B189] Schaeffer S. Harper A. Raja R. Jaiswal P. Dhingra A. (2014). Comparative analysis of predicted plastid-targeted proteomes of sequenced higher plant genomes. PLoS One 9, e112870. 10.1371/journal.pone.0112870 25393533 PMC4231079

[B190] Schaeffer S. M. Christian R. Castro-Velasquez N. Hyden B. Lynch-Holm V. Dhingra A. (2017). Comparative ultrastructure of fruit plastids in three genetically diverse genotypes of apple (Malus × domestica Borkh.) during development. Plant Cell. Rep. 36, 1627–1640. 10.1007/s00299-017-2179-z 28698906 PMC5693628

[B191] Schnell D. J. Kessler F. Blobel G. (1994). Isolation of components of the chloroplast protein import machinery. Sci. (1979) 266, 1007–1012. 10.1126/science.7973649 7973649

[B192] Schweiger R. Soll J. Jung K. Heermann R. Schwenkert S. (2013). Quantification of interaction strengths between chaperones and tetratricopeptide repeat domain-containing membrane proteins. J. Biol. Chem. 288, 30614–30625. 10.1074/jbc.M113.493015 24036116 PMC3798532

[B193] Seedorf M. Soll J. (1995). Copper chloride, an inhibitor of protein import into chloroplasts. FEBS Lett. 367, 19–22. 10.1016/0014-5793(95)00529-I 7601278

[B194] Seedorf M. Waegemann K. Soll J. (1995). A constituent of the chloroplast import complex represents a new type of GTP-binding protein. Plant J. 7, 401–411. 10.1046/j.1365-313X.1995.7030401.x 7757113

[B195] Selosse M.-A. Albert B. Godelle B. (2001). Reducing the genome size of organelles favours gene transfer to the nucleus. Trends Ecol. Evol. 16, 135–141. 10.1016/S0169-5347(00)02084-X 11179577

[B196] Shanmugabalaji V. Kessler F. (2019). CHLORAD: Eradicating translocon components from the outer membrane of the chloroplast. Mol. Plant 12, 467–469. 10.1016/j.molp.2019.03.002 30890496

[B197] Sheiner L. Vaidya A. B. McFadden G. I. (2013). The metabolic roles of the endosymbiotic organelles of Toxoplasma and Plasmodium spp. Curr. Opin. Microbiol. 16, 452–458. 10.1016/j.mib.2013.07.003 23927894 PMC3767399

[B198] Shi L. X. Theg S. M. (2010). A stromal heat shock protein 70 system functions in proteinimport into chloroplasts in the moss physcomitrella patens. Plant Cell. 22, 205–220. 10.1105/tpc.109.071464 20061551 PMC2828695

[B199] Simkin A. J. Kapoor L. Doss C. G. P. Hofmann T. A. Lawson T. Ramamoorthy S. (2022). The role of photosynthesis related pigments in light harvesting, photoprotection and enhancement of photosynthetic yield in planta. Photosynth Res. 152, 23–42. 10.1007/s11120-021-00892-6 35064531

[B200] Singer A. Poschmann G. Mühlich C. Valadez-Cano C. Hänsch S. Hüren V. (2017). Massive protein import into the early-evolutionary-stage photosynthetic organelle of the amoeba Paulinella chromatophora. Curr. Biol. 27, 2763–2773.e5. 10.1016/j.cub.2017.08.010 28889978

[B201] Sjuts I. Soll J. Bölter B. (2017). Import of soluble proteins into chloroplasts and potential regulatory mechanisms. Front. Plant Sci. 8, 168. 10.3389/fpls.2017.00168 28228773 PMC5296341

[B202] Smeekens S. Bauerle C. Hageman J. Keegstra K. Weisbeek P. (1986). The role of the transit peptide in the routing of precursors toward different chloroplast compartments. Cell. 46, 365–375. 10.1016/0092-8674(86)90657-4 3731274

[B203] Smith M. D. Rounds C. M. Wang F. Chen K. Afitlhile M. Schnell D. J. (2004). atToc159 is a selective transit peptide receptor for the import of nucleus-encoded chloroplast proteins. J. Cell. Biol. 165, 323–334. 10.1083/jcb.200311074 15138290 PMC2172197

[B204] Sohrt K. Soll J. (2000). Toc64, a new component of the protein translocon of chloroplasts. J. Cell. Biol. 148, 1213–1221. 10.1083/jcb.148.6.1213 10725334 PMC2174300

[B205] Solymosi K. Keresztes A. (2013). Plastid structure, diversification and interconversions II. Land plants. Curr. Chem. Biol. 6, 187–204. 10.2174/2212796811206030003

[B206] Sommer M. Rudolf M. Tillmann B. Tripp J. Sommer M. S. Schleiff E. (2013). Toc33 and Toc64-III cooperate in precursor protein import into the chloroplasts of *Arabidopsis thaliana* . Plant Cell. Environ. 36, 970–983. 10.1111/pce.12030 23131143

[B207] Soon F. F. Ng L. M. Zhou X. E. West G. M. Kovach A. Tan M. H. E. (2012). Molecular mimicry regulates ABA signaling by SnRK2 kinases and PP2C phosphatases. Sci. (1979) 335, 85–88. 10.1126/science.1215106 PMC3584687 22116026

[B208] Sperschneider J. Catanzariti A.-M. DeBoer K. Petre B. Gardiner D. M. Singh K. B. (2017). LOCALIZER: Subcellular localization prediction of both plant and effector proteins in the plant cell. Sci. Rep. 7, 44598. 10.1038/srep44598 28300209 PMC5353544

[B209] Stahl T. Glockmann C. Soll J. Heins L. (1999). Tic40, a new “old” subunit of the chloroplast protein import translocon. J. Biol. Chem. 274, 37467–37472. 10.1074/jbc.274.52.37467 10601321

[B210] Stegemann S. Hartmann S. Ruf S. Bock R. (2003). High-frequency gene transfer from the chloroplast genome to the nucleus. Proc. Natl. Acad. Sci. 100, 8828–8833. 10.1073/pnas.1430924100 12817081 PMC166398

[B211] Stengel A. Benz J. P. Buchanan B. B. Soll J. Bölter B. (2009). Preprotein import into chloroplasts via the toc and tic complexes is regulated by redox signals in pisum sativum. Mol. Plant 2, 1181–1197. 10.1093/mp/ssp043 19995724

[B212] Stengel A. Benz P. Balsera M. Soll J. Bölter B. (2008). TIC62 redox-regulated translocon composition and dynamics. J. Biol. Chem. 283, 6656–6667. 10.1074/jbc.M706719200 18180301

[B213] Su P. H. Li H. M. (2010). Stromal Hsp70 is important for protein translocation into pea and Arabidopsis chloroplasts. Plant Cell. 22, 1516–1531. 10.1105/tpc.109.071415 20484004 PMC2899880

[B214] Sugiura M. (1992). The chloroplast genome. Plant Mol. Biol. 19, 149–168. 10.1007/BF00015612 1600166

[B215] Sun Q. Zybailov B. Majeran W. Friso G. Olinares P. D. B. van Wijk K. J. (2009). PPDB, the plant proteomics database at cornell. Nucleic Acids Res. 37, 969–974. 10.1093/nar/gkn654 PMC2686560 18832363

[B216] Sun Y. J. Forouhar F. Li H. M. Tu S. L. Yeh Y. H. Kao S. (2002). Crystal structure of pea toc34, a novel gtpase of the chloroplast protein translocon. Nat. Struct. Biol. 9, 95–100. 10.1038/nsb744 11753431

[B217] Sun Y. Yao Z. Ye Y. Fang J. Chen H. Lyu Y. (2022). Ubiquitin-based pathway acts inside chloroplasts to regulate photosynthesis. Sci. Adv. 8, eabq7352. 10.1126/sciadv.abq7352 36383657 PMC9668298

[B218] Suzuki M. Takahashi S. Kondo T. Dohra H. Ito Y. Kiriiwa Y. (2015). Plastid proteomic analysis in tomato fruit development. PLoS One 10, 01372666 10.1371/journal.pone.0137266 PMC4570674 26371478

[B219] Sveshnikova N. Soll J. Schleiff E. (2000). Toc34 is a preprotein receptor regulated by GTP and phosphorylation. Proc. Natl. Acad. Sci. U. S. A. 97, 4973–4978. 10.1073/pnas.080491597 10781107 PMC18342

[B220] Teixeira P. F. Kmiec B. Branca R. M. M. Murcha M. W. Byzia A. Ivanova A. (2017). A multi-step peptidolytic cascade for amino acid recovery in chloroplasts. Nat. Chem. Biol. 13, 15–17. 10.1038/nchembio.2227 27820795

[B221] Teng Y. S. Chan P. T. Li H. M. (2012). Differential age-dependent import regulation by signal peptides. PLoS Biol. 10, e1001416. 10.1371/journal.pbio.1001416 23118617 PMC3484058

[B222] Teng Y. S. Su Y. S. Chen L. J. Lee Y. J. Hwang I. Li H. M. (2006). Tic21 is an essential translocon component for protein translocation across the chloroplast inner envelope membrane. Plant Cell. 18, 2247–2257. 10.1105/tpc.106.044305 16891400 PMC1560916

[B223] Teresinski H. J. Gidda S. K. Nguyen T. N. D. Howard N. J. M. Porter B. K. Grimberg N. (2019). An RK/ST C-terminal motif is required for targeting of OEP7.2 and a subset of other arabidopsis tail-anchored proteins to the plastid outer envelope membrane. Plant Cell. Physiol. 60, 516–537. 10.1093/pcp/pcy234 30521026

[B224] Tsai J. Y. Chu C. C. Yeh Y. H. Chen L. J. Li H. M. Hsiao C. D. (2013). Structural characterizations of the chloroplast translocon protein Tic110. Plant J. 75, 847–857. 10.1111/tpj.12249 23711301 PMC3823011

[B225] Tu S. Chen L. Smith M. D. Su Y. Schnell D. J. Li H. (2004). Import pathways of chloroplast interior proteins and the outer-membrane protein OEP14 converge at Toc75. Plant Cell. 16, 2078–2088. 10.1105/tpc.104.023952 15258267 PMC519199

[B226] Tuba Z. Lichtenthaler H. K. Csintalan Z. Nagy Z. Szente K. (1994). Reconstitution of chlorophylls and photosynthetic CO2 assimilation upon rehydration of the desiccated poikilochlorophyllous plant Xerophyta scabrida (Pax) Th. Dur. et Schinz. Planta 192, 414–420. 10.1007/BF00198578

[B227] Van ’t Hof R. de Kruijff B. (1995). Transit sequence-dependent binding of the chloroplast precursor protein ferredoxin to lipid vesicles and its implications for membrane stability. FEBS Lett. 361, 35–40. 10.1016/0014-5793(95)00135-V 7890037

[B228] van Wijk K. J. Baginsky S. (2011). Plastid proteomics in higher plants: Current state and future goals. Plant Physiol. 155, 1578–1588. 10.1104/pp.111.172932 21350036 PMC3091083

[B229] van Wijk K. J. (2015). Protein maturation and proteolysis in plant plastids, mitochondria, and peroxisomes. Annu. Rev. Plant Biol. 66, 75–111. 10.1146/annurev-arplant-043014-115547 25580835

[B230] Viana A. A. B. Li M. Schnell D. J. (2010). Determinants for stop-transfer and post-import pathways for protein targeting to the chloroplast inner envelope membrane. J. Biol. Chem. 285, 12948–12960. 10.1074/jbc.M110.109744 20194502 PMC2857071

[B231] Villarejo A. Burén S. Larsson S. Déjardin A. Monné M. Rudhe C. (2005). Evidence for a protein transported through the secretory pathway en route to the higher plant chloroplast. Nat. Cell. Biol. 7, 1224–1231. 10.1038/ncb1330 16284624

[B232] von Heijne G. Steppuhn J. Herrmann R. G. (1989). Domain structure of mitochondrial and chloroplast targeting peptides. Eur. J. Biochem. 180, 535–545. 10.1111/j.1432-1033.1989.tb14679.x 2653818

[B233] von Zychlinski A. Kleffman T. Krishnamurthy N. Sjölander K. Baginsky S. Gruissem W. (2005). Proteome analysis of the rice etioplast: Metabolic and regulatory networks and novel protein functions. Mol. Cell. Proteomics 4, 1072–1084. 10.1074/mcp.M500018-MCP200 15901827

[B234] Wang F. Agne B. Kessler F. Schnell D. J. (2008). The role of GTP binding and hydrolysis at the atToc159 preprotein receptor during protein import into chloroplasts. J. Cell. Biol. 183, 87–99. 10.1083/jcb.200803034 18824565 PMC2557045

[B235] Wang P. Xue L. Batelli G. Lee S. Hou Y. J. Van Oosten M. J. (2013). Quantitative phosphoproteomics identifies SnRK2 protein kinase substrates and reveals the effectors of abscisic acid action. Proc. Natl. Acad. Sci. U. S. A. 110, 11205–11210. 10.1073/pnas.1308974110 23776212 PMC3703982

[B236] Whatley J. M. (1985). Chromoplasts in some cycads. New Phytol. 101, 595–604. 10.1111/j.1469-8137.1985.tb02865.x

[B237] Wieprecht T. Apostolov O. Beyermann M. Seelig J. (2000). Interaction of a mitochondrial presequence with lipid membranes: Role of helix formation for membrane binding and perturbation. Biochemistry 39, 15297–15305. 10.1021/bi001774v 11112515

[B238] Wiesemann K. Simm S. Mirus O. Ladig R. Schleiff E. (2019). Regulation of two GTPases Toc159 and Toc34 in the translocon of the outer envelope of chloroplasts. Biochimica Biophysica Acta (BBA) - Proteins Proteomics 1867, 627–636. 10.1016/j.bbapap.2019.01.002 30611779

[B239] Wimmer D. Bohnhorst P. Shekhar V. Hwang I. Offermann S. (2017). Transit peptide elements mediate selective protein targeting to two different types of chloroplasts in the single-cell C4 species Bienertia sinuspersici. Sci. Rep. 7, 41187. 10.1038/srep41187 28112241 PMC5253730

[B240] Wise R. (2006). “The diversity of plastid form and function,” in The structure and function of plastids. Editors Wise R. R. Hoober J. K. (Dordrecht: Springer), 3–26. 10.1007/978-1-4020-4061-0_1

[B241] Yeh Y. H. Kesavulu M. M. Li H. M. Wu S. Z. Sun Y. J. Konozy E. H. E. (2007). Dimerization is important for the GTPase activity of chloroplast translocon components atToc33 and psToc159. J. Biol. Chem. 282, 13845–13853. 10.1074/jbc.M608385200 17337454

[B242] Yoon H. S. Hackett J. D. Ciniglia C. Pinto G. Bhattacharya D. (2004). A molecular timeline for the origin of photosynthetic eukaryotes. Mol. Biol. Evol. 21, 809–818. 10.1093/molbev/msh075 14963099

[B243] Zeng Y. Pan Z. Ding Y. Zhu A. Cao H. Xu Q. (2011). A proteomic analysis of the chromoplasts isolated from sweet orange fruits [Citrus sinensis (L.) Osbeck]. J. Exp. Bot. 62, 5297–5309. 10.1093/jxb/err140 21841170 PMC3223033

[B244] Zhang R. Nowack E. C. M. Price D. C. Bhattacharya D. Grossman A. R. (2017). Impact of light intensity and quality on chromatophore and nuclear gene expression in Paulinella chromatophora, an amoeba with nascent photosynthetic organelles. Plant J. 90, 221–234. 10.1111/tpj.13488 28182317

[B245] Zhang X. P. Glaser E. (2002). Interaction of plant mitochondrial and chloroplast signal peptides with the Hsp70 molecular chaperone. Trends Plant Sci. 7, 14–21. 10.1016/S1360-1385(01)02180-X 11804822

[B246] Zhang Y. Xiao G. Wang X. Zhang X. Friml J. (2019). Evolution of fast root gravitropism in seed plants. Nat. Commun. 10, 3480. 10.1038/s41467-019-11471-8 31375675 PMC6677796

[B247] Zhong R. Thompson J. Ottesen E. Lamppa G. K. (2010). A forward genetic screen to explore chloroplast protein import *in vivo* identifies Moco sulfurase, pivotal for ABA and IAA biosynthesis and purine turnover. Plant J. 63, 44–59. 10.1111/j.1365-313X.2010.04220.x 20374530

[B248] Zhong R. Wan J. Jin R. Lamppa G. (2003). A pea antisense gene for the chloroplast stromal processing peptidase yields seedling lethals in Arabidopsis: Survivors show defective GFP import *in vivo* . Plant J. 34, 802–812. 10.1046/j.1365-313X.2003.01772.x 12795700

[B249] Zhu M. Lin J. Ye J. Wang R. Yang C. Gong J. (2018). A comprehensive proteomic analysis of elaioplasts from citrus fruits reveals insights into elaioplast biogenesis and function. Hortic. Res. 5, 6–10. 10.1038/s41438-017-0014-x 29423236 PMC5802726

[B250] Zimorski V. Ku C. Martin W. F. Gould S. B. (2014). Endosymbiotic theory for organelle origins. Curr. Opin. Microbiol. 22, 38–48. 10.1016/j.mib.2014.09.008 25306530

[B251] Zufferey M. Montandon C. Douet V. Demarsy E. Agne B. Baginsky S. (2017). The novel chloroplast outer membrane kinase KOC1 is a required component of the plastid protein import machinery. J. Biol. Chem. 292, 6952–6964. 10.1074/jbc.M117.776468 28283569 PMC5409464

[B252] Zybailov B. Rutschow H. Friso G. Rudella A. Emanuelsson O. Sun Q. (2008). Sorting signals, N-terminal modifications and abundance of the chloroplast proteome. PLoS One 3, e1994. 10.1371/journal.pone.0001994 18431481 PMC2291561

